# BIANCA (Brain Intensity AbNormality Classification Algorithm): A new tool for automated segmentation of white matter hyperintensities

**DOI:** 10.1016/j.neuroimage.2016.07.018

**Published:** 2016-11-01

**Authors:** Ludovica Griffanti, Giovanna Zamboni, Aamira Khan, Linxin Li, Guendalina Bonifacio, Vaanathi Sundaresan, Ursula G. Schulz, Wilhelm Kuker, Marco Battaglini, Peter M. Rothwell, Mark Jenkinson

**Affiliations:** a Centre for the Functional MRI of the Brain (FMRIB), Nuffield Department of Clinical Neurosciences, University of Oxford, UK; b Centre for Prevention of Stroke and Dementia, Nuffield Department of Clinical Neurosciences, University of Oxford, UK; c Department of Medicine, Surgery and Neuroscience, University of Siena, Siena, Italy

**Keywords:** White matter hyperintensities, Automated segmentation, Brain MRI, Neurodegeneration, Vascular pathology

## Abstract

Reliable quantification of white matter hyperintensities of presumed vascular origin (WMHs) is increasingly needed, given the presence of these MRI findings in patients with several neurological and vascular disorders, as well as in elderly healthy subjects.

We present BIANCA (Brain Intensity AbNormality Classification Algorithm), a fully automated, supervised method for WMH detection, based on the k-nearest neighbour (*k*-NN) algorithm. Relative to previous *k*-NN based segmentation methods, BIANCA offers different options for weighting the spatial information, local spatial intensity averaging, and different options for the choice of the number and location of the training points. BIANCA is multimodal and highly flexible so that the user can adapt the tool to their protocol and specific needs.

We optimised and validated BIANCA on two datasets with different MRI protocols and patient populations (a “predominantly neurodegenerative” and a “predominantly vascular” cohort).

BIANCA was first optimised on a subset of images for each dataset in terms of overlap and volumetric agreement with a manually segmented WMH mask. The correlation between the volumes extracted with BIANCA (using the optimised set of options), the volumes extracted from the manual masks and visual ratings showed that BIANCA is a valid alternative to manual segmentation. The optimised set of options was then applied to the whole cohorts and the resulting WMH volume estimates showed good correlations with visual ratings and with age. Finally, we performed a reproducibility test, to evaluate the robustness of BIANCA, and compared BIANCA performance against existing methods.

Our findings suggest that BIANCA, which will be freely available as part of the FSL package, is a reliable method for automated WMH segmentation in large cross-sectional cohort studies.

## Highlights

• BIANCA is a new tool for automated segmentation of white matter hyperintensities.
• BIANCA is multimodal, flexible, computationally lean, robust, freely available.
• We optimised and validated BIANCA on two different MRI protocols and populations.
• WMH volumes derived with BIANCA showed good correlations with visual ratings and age.
• BIANCA is promising for application in large cross-sectional cohort studies.

## Introduction

White matter hyperintensities of presumed vascular origin (WMHs), also known as leukoariosis, white matter lesions, or white matter disease (Wardlaw et al., 2013), are common findings in MRI scans and appear hyperintense on T2-weighted, fluid attenuated inversion recovery (FLAIR), and proton density-weighted images. WMHs are common in patients with cardiovascular risk factors and symptomatic cerebrovascular disease (Li et al., 2013, Simoni et al., 2012), and are associated with increased risk of functional decline, dementia, and death (de Groot et al., 2000, Debette and Markus, 2010, Inzitari et al., 2009, Jeerakathil et al., 2004, Longstreth et al., 1996, Pantoni et al., 2005, Prins and Scheltens, 2015, van Dijk et al., 2002). However, they are also increasingly found in healthy elderly on MRI scans performed in routine clinical practice, as brain MRI is the recommended investigation for most neurological conditions affecting older adults. Therefore there is a need to improve the quantification of WMH in order to facilitate studies to better clarify their diagnostic and prognostic value in both healthy and diseased populations. The characterization of WMH in terms of volume, location and number of lesions (assessed either with visual rating scales or quantitative measurements) has also been recently included in the standards for reporting vascular changes on neuroimaging, which have been formulated for research studies, but are also applicable in clinical settings (Wardlaw et al., 2013).

The most common visual rating scales used to assess WMH are the Fazekas scale (Fazekas et al., 1987), the Scheltens scale (Scheltens et al., 1993), and the age-related white matter changes (ARWMC) scale (Wahlund et al., 2001). They are frequently used in clinical settings, but are also still used in research studies (Kreisel et al., 2013, Simoni et al., 2012). However visual rating is time consuming, suffers from intra- and inter-operator variability, potentially leading to inconsistencies among studies, is potentially subject to observer bias, and only provides discrete measurements of WMH (Mantyla et al., 1997). In addition, methods based on visual ratings do not provide precise information about the spatial localization of WMH. Being able to provide the exact localization of WMH at the voxel level is important, as it can be used to obtain a better association between WMH and specific symptoms, or to better define patterns related to normal versus pathological ageing (Benson et al., 2002, Biesbroek et al., 2013, Duering et al., 2014, Rostrup et al., 2012, Smith et al., 2000). Voxel-wise WMH maps can also be used as a nuisance variable in analyses of other imaging modalities, to disentangle the contribution of WMH from other MRI abnormalities.

Therefore, a method that is objective, automated, and that provides quantitative measures and the exact localization of WMH at the voxel level is highly desirable.

The assessment of WMH with quantitative volumetric measurements is much more used in research settings. Several methods have been developed, mostly in-house. However, despite the number of proposed methods, none of the algorithms is currently widely used and only very few of them are publicly available (Damangir et al., 2012, Lao et al., 2008, Schmidt et al., 2012). Several automated and voxel-wise methods have been developed for the detection of multiple sclerosis (MS) lesions (Mortazavi et al., 2012). However, boundaries of MS lesions are often sharper than those of WMH and WMH patterns are very heterogeneous, ranging from punctuate lesions in the deep white matter to large confluent periventricular lesions. For WMH, several segmentation algorithms exist (Admiraal-Behloul et al., 2005, Anbeek et al., 2004, de Boer et al., 2009, DeCarli et al., 1995, Dyrby et al., 2008, Ramirez et al., 2011), and a recent review by Caligiuri and colleagues (Caligiuri et al., 2015) compared different existing algorithms. Besides the inherent difficulty of the problem, most of the algorithms have been validated on small samples leading to over fitting, are not freely available or easily accessible, or, being developed to be protocol and/or study specific, may not be able to work outside a very limited protocol. The need for an automated tool that is multimodal, flexible, freely available and well supported is made even more important by the growing number of large cross-sectional cohort studies: the OXVASC study (Rothwell et al., 2004) with a target of 1500 subjects in its phase 3 (OxVASC-Cog 3 – 2012–2017), the Whitehall study (Filippini et al., 2014) with 800 subjects, and the UK Biobank study (http://imaging.ukbiobank.ac.uk), with 100,000 subjects, are just a few examples. Given the importance of investigating WMH in ageing and diseased populations to identify biomarkers and understand ageing/disease processes, these cohort studies would definitively benefit from a flexible, automated method, as it would not be feasible performing WMH segmentation manually.

Here we present BIANCA (Brain Intensity AbNormality Classification Algorithm), a fully automated, supervised method for WMH detection, that uses the k-nearest neighbour (k-NN) algorithm (see Algorithm overview for details). Anbeek and colleagues (Anbeek et al., 2004) previously presented a method for automatic segmentation of WMHs based on the *k*-NN classification technique using information from different MRI modalities (T1-weighted, inversion recovery, proton density-weighted, T2-weighted and fluid attenuation inversion recovery - FLAIR). They also included spatial information and quantitatively validated the algorithm, on a voxel basis, using 20 subjects with arterial vascular disease. Steenwijk and colleagues (Steenwijk et al., 2013) further investigated different approaches for intensity features normalization and introduced the use of tissue priors. They tested the algorithm on 20 patients with MS, 16 healthy controls, and performed a validation on an independent set of 20 subjects with hypertension.

BIANCA relies on a similar approach to the ones used in the above mentioned studies, using the *k*-NN algorithm, with flexible features (MRI modalities and spatial features) but introducing different options like the possibility of weighting the spatial coordinates, using local spatial intensity averaging (the “patch” option – see BIANCA options for details) and changing the number and location of the training points.

In this paper we optimised BIANCA on two datasets that were different in terms of patient populations and MRI protocol (see Test datasets for details). These two large datasets are representative of groups of patients where the clinical importance of WMH is being increasingly recognized: a “predominantly neurodegenerative” cohort including people with, or at risk of, Alzheimer's disease (AD) and a “predominantly vascular” cohort including people with, or at risk of, vascular cognitive impairment.

Given the absence of a gold standard for assessing WMH segmentation, we evaluated BIANCA performance with multiple methods both in the optimization and validation phase. In the optimization phase, the performance of BIANCA was evaluated on a subsample of subjects for each dataset both in terms of overlap and volumetric agreement with manual segmentations. The volumes extracted with BIANCA using the optimised set of options were then correlated with the volumes extracted from the manual masks and with visual ratings. In the validation phase, the measurements of WMH volume derived from BIANCA were evaluated by correlation with visual ratings and age. Finally, we performed a reproducibility test, to evaluate the robustness of BIANCA and compared BIANCA performance against existing methods. The tool will be freely available and included in the next release of FSL (FMRIB software library).

## Materials and methods

### Test datasets

This section describes the datasets used to optimise and validate BIANCA for the detection of white matter hyperintensities of presumed vascular origin (Wardlaw et al., 2013). The datasets are different in terms of populations, were acquired on different scanners and using different imaging protocols (see details below).

Exclusion criteria applied to both cohorts for the purposes of the present study were: presence of intracranial haemorrhage; intracranial space occupying lesion; WMH mimics (multiple sclerosis and irradiation induced gliosis); brain defect due to previous neurosurgery or developmental anomalies; large chronic, subacute or acute infarcts (i.e., > 2 cm on either T1-, T2-weighted or DWI sequences); significant movement artefacts.

For both datasets WMHs were graded on FLAIR images by a trained operator (L.L.) who provided visual ratings according to the following scales: 1) a modified version of the Fazekas scale (Fazekas et al., 1987), considering periventricular and deep white matter lesions altogether (range total score 0–6); 2) the ARWMC (Age-Related White Matter Changes, (Wahlund et al., 2001)) scale, rating 5 different regions (frontal, parieto-occipital, temporal, basal ganglia, infratentorial) in both hemispheres according to a 0–3 score (range total score 0–30).

#### Dataset 1 (neurodegenerative cohort)

MRI data from 85 older adults (25 with probable Alzheimer's Disease - AD, 24 with amnestic mild cognitive impairment - MCI, 11 with subjective cognitive impairment and 25 cognitively healthy control subjects - HC) recruited from the Oxford Project to Investigate Memory and Ageing (OPTIMA) and from the Memory Assessment Clinic at the John Radcliffe Hospital in Oxford (Zamboni et al., 2013) were included in the “neurodegenerative cohort” (age range 57–91 years, mean age 75 ± 7 years, F:M = 39:46).

MRI images were acquired at the University of Oxford OCMR centre on a 3 T Siemens Trio scanner using a T2-weighted, fluid-attenuated inversion recovery (FLAIR) research sequence (TR/TE = 9000/89 ms, flip angle 150^o^, FOV 220 mm, voxel size 1.1 × 0.9 × 3 mm). The visual ratings according to the Fazekas score had a range of 0 to 6 (mean ± sd = 2.6 ± 1.4) and with the ARWMC score from 0 to 24 (mean ± sd = 6.1 ± 5.3).

High-resolution T1-weighted images (3D MP-RAGE) were also acquired (TR/TE = 2040/4.7 ms, flip angle 8^o^, FOV 192 mm, voxel size 1 mm isotropic).

#### Dataset 2 (vascular cohort)

MRI data from 474 consecutive eligible participants in the Oxford Vascular Study (OXVASC, (Rothwell et al., 2004)) who had recently experienced a minor non-disabling stroke or transient ischemic attack (TIA) were included in the “vascular cohort” (age range 20–102 years, mean age 67.4 ± 14.3 years, F:M = 240:234).

Scanning was performed at the Oxford Acute Vascular Imaging Centre (AVIC) on a 3 T Siemens Verio scanner using a T2-weighted, FLAIR clinical sequence (TR/TE = 9000/94.0 ms, flip angle 150^o^, FOV 200 mm, matrix size 464 × 28 × 512, voxel size 0.8 × 5 × 0.8 mm). The WMH visual ratings according to the Fazekas scale varied from 0 to 6 (mean ± sd = 1.9 ± 1.8) and with the ARWMC scale from 0 to 24 (mean ± sd = 3.9 ± 4.8).

Twenty participants (age range 40–91 years, mean age 68 ± 13 years, F:M = 9:11) were re-scanned after 2 weeks to 28 months on the same scanner, using the same protocol. The severity of WMH assessed with visual ratings (categorical ARWMC: 0 = No, 1–5 = mild, 6 = 10 mod, > 10 severe) was not different from the first scan, so we used those data to test the reproducibility of BIANCA (see Reproducibility test for details).

### Brain Intensity AbNormality Classification Algorithm (BIANCA)

#### Algorithm overview

The *k*-NN algorithm is a method for classifying objects based on the closest training examples in the feature space. An object is classified by a majority vote of its neighbours in the feature space, with the object being assigned to the class most common among its *k* nearest neighbours. The proportion of the votes for the winning class is returned, so that *k*-NN's output is probabilistic.

When applying *k*-NN to the problem of WMH segmentation, each axis of the feature space represents one of the voxel's features. In BIANCA, the feature space includes both intensity and spatial features (details of the different features tested are described in BIANCA options). The algorithm requires a training set with pre-classified voxels (i.e. manually segmented images) that is used to create a set of feature vectors for WMH and non-WMH classes, where each voxel selected from the training set generates one feature vector. In this study, we tested the importance of the selection process in the training dataset by comparing results generated using different numbers of training points for the two classes and different locations for the selection of non-WMH voxels, as well as the inclusion of subjects with different WMH load in the training dataset (see BIANCA options). The selected training voxels are then used to generate feature vectors, and the classification of a voxel belonging to a new subject's image is performed by forming a feature vector, adding it to the feature space, and then looking at the *k* training feature vectors that are closest to it. Steenwijk and colleagues (Steenwijk et al., 2013) used *k* = 40 and, after testing other values, confirmed that *k* in that range is suitable for this type of segmentation problems. We therefore decided to set *k* to 40 in the current study, which we have found gives good performance. The output of the classification step is the probability of a voxel of being WMH, calculated as the proportion of *k* neighbours belonging to the WMH class. Finally, in the post-processing step (see *Post-processing options*), if the proportion of *k* neighbours belonging to the WMH class exceeds a certain threshold, and if the voxel is located in the white matter, the voxel is classified as WMH.

#### Generation of a training dataset

WMHs of 21 subjects of Dataset 1 and 109 subjects of Dataset 2 were manually segmented on FLAIR images, producing binary masks with the value of 0 (non-WMH class) or 1 (WMH class). The manual segmentation was achieved through a consensus among three trained operators (G.Z., A.K., G.B.), who also had access to T1w images for subjects in Dataset 1.

**Fig. S1:**
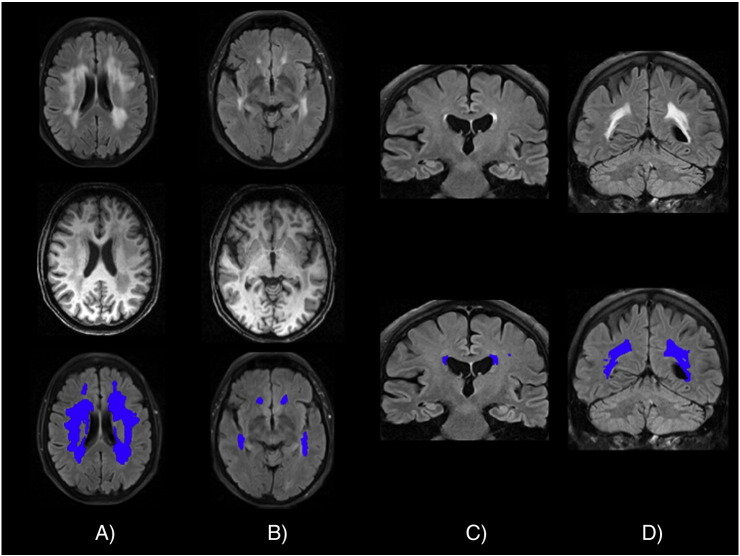
Examples of manual masks for different datasets (a,b Dataset 1; c,d Dataset 2) and WMH load (a,c low WMH load; b,d high WMH). Top row shows the FLAIR images, the bottom row shows the manual mask overlaid in blue. For Dataset 1, the corresponding T1w image is shown in the middle row.

Examples of manual masks for the two datasets are shown in Supplementary Fig. S1. These manually segmented masks were used both to train BIANCA and to judge its performance by comparing BIANCA output and the manual masks in leave-one-out tests (see BIANCA optimisation for details). To avoid biased results, and to be able to also test the accuracy of BIANCA in segmenting the WMH for the subjects included in the training dataset, BIANCA automatically applies the leave-one-out cross-validation method: a reduced training set is used for the segmentation of a subject from the training dataset, where the reduced training set excludes this subject and is built from the voxels of the remaining training subjects.

#### BIANCA options

**Fig. 1:**
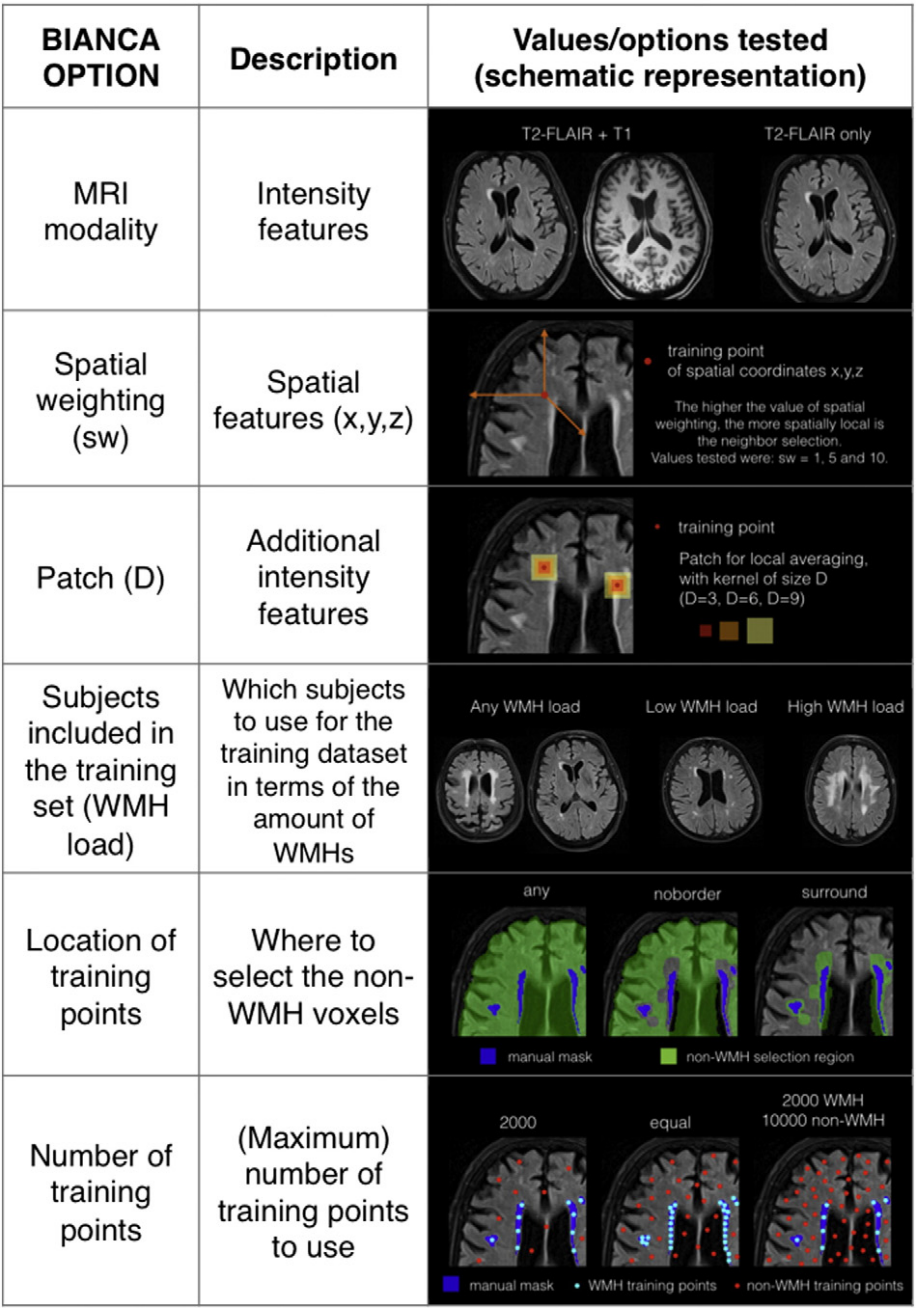
BIANCA options. List and brief description of the different options available with BIANCA tool and schematic representation of the different values tested in this study during the phase of algorithm optimisation. See main text for further details.

This section describes the different options that are currently available in BIANCA and have been tested in this work. Fig. 1 shows a schematic representation of the options and the set of values/parameters tested (for a full description of the tests, see BIANCA optimisation).

##### • Multiple MRI modalities

BIANCA can include any set of MRI modalities from either 2D or 3D acquisitions, from which the intensity features are extracted. BIANCA works with images in the subject's space, but they need to be registered to a consistent reference MRI modality. BIANCA is flexible also in terms of reference modality, and the choice could depend, for example, on the image quality and the aim of the study: T1 images are usually the ones with the highest resolution, while FLAIR images are the ones with highest contrast for WMH and usually used to create the manual masks, but have lower resolution. In this study we decided to use T1 images as reference for the main analyses on Dataset 1, to avoid down-sampling it when registering it to the FLAIR image, however, we tested a subset of options also in FLAIR space (see supplementary material for details). Registrations between the two modalities were performed using FLIRT (Jenkinson et al., 2002, Jenkinson and Smith, 2001) with trilinear interpolation (the manual masks was thresholded at 0.25 after registration to T1 to compensate for interpolation). Intensity normalization using variance scaling (Anbeek et al., 2004, Steenwijk et al., 2013) is automatically applied by BIANCA to all images.

##### • Spatial weighting (sw)

BIANCA can also utilise spatial coordinates, formed by using a linear registration (with FLIRT) to find each voxel's corresponding MNI coordinate (x,y,z). Anbeek and colleagues (Anbeek et al., 2004) already demonstrated that information about the coordinates of a voxel increases the accuracy of the segmentation, as in some regions of the brain WMH are more likely to occur than in others. The spatial weighting option takes this further and applies a linear scale factor, after the normalization of the feature vector, to the coordinate data within the feature vector. This scaling provides a way of emphasizing (or de-emphasizing) the role that the coordinates play, with a higher value for spatial weighting leading to the neighbouring feature vectors being more likely to come from similar spatial locations, effectively making the *k*-NN method use more local training data. This approach works because with a high spatial weighting, even a relatively small difference between two voxels in the MNI space will make them very far away from each other in the feature space, and therefore, only nearest neighbours with very similar spatial coordinates will be selected for the classification. If sw = 1 (the default) the data is simply variance normalised, whereas if sw = 0 the spatial coordinates will be ignored, and if sw becomes very large then the nearest neighbour selection would effectively ignore the intensity features and the output would become a prior lesion probability map, based purely on lesion locations in the training dataset. For this option to be used, a transformation matrix from the subject space to standard MNI space is required.

##### • Patch

Additional intensity features, containing the local average intensity for each modality, can also be included using the “patch” option. One or more patch sizes can be chosen, by setting the size (D – in voxels) of the square/cubic kernel used for local averaging. The inclusion of intensity information about a small neighbourhood of each voxel has been proposed before (Dyrby et al., 2008, Lao et al., 2008), in order to make the segmentation more robust to misregistration. The patches used for local averaging with BIANCA can be 2D or 3D. In this study we tested a 3D patch on Dataset 1 and 2D patch on Dataset 2, due to the highly anisotropic voxels. When calculating the local average for a voxel on the border of the brain mask, the local averaging is performed by averaging only the voxels within the kernel that are inside the brain mask.

##### • Subjects included in the training set (WMH load)

We tested whether BIANCA's performance would change when changing the subjects used in the training dataset according to the amount of WMHs, as judged by the visual ratings provided. We compared three options: using all the subjects for which we had manual segmentation available (“any WMH load” option), using only those with the highest WMH load (“high WMH load” option) or using those with the lowest WMH load (“low WMH load” option). In all cases, when running the segmentation on an image included in the training dataset, BIANCA automatically excludes that subject from the training dataset (leave-one-out method).

##### • Location of non-WMH training points

By default BIANCA will use, as non-WMH points, training points inside the brain that are not classified as WMH in the manual masks supplied (this is the “any” option in this study). There are also options to restrict the selection so that points close to the edge of the WMH-labelled voxels are preferentially selected as non-WMH points (“surround” option), or conversely, excluded these nearby voxels from the training set (“no border” option). The rationale behind these options is to test if, and how, information around the WMHs' edges is important for the segmentation.

##### • Number of training points

BIANCA has three options for selecting the number of training points for WMH and non-WMH voxels within the manual WMH masks supplied:

- Fixed + Equal (FE) number: by setting a fixed value N (in this study set to 2000 voxels for each subject included in the training dataset), BIANCA will use up to N points per subject classified as WMH (limited by the number of points present in the manual masks) and the same number of non-WMH points.
- All WMH + Equal (AE) number: BIANCA will use, for each subject included in the training dataset, all the points classified as WMH in the manual masks and an equal number of points classified as non-WMH.
- Fixed + Unbalanced (FU) number: it is possible to specify different numbers of training points for WMH and non-WMH. In this study we initially used 2000 points per subject for WMH (capped by the number of points available in the manual masks) and 10,000 per subject for non-WMH. In a second phase of BIANCA optimisation (see BIANCA optimization and supplementary material) we also tested the use of more training points either maintaining the same ratio (1:5) or increasing the number of non-WMH points (up to 1:29 ratio).

#### Post-processing options

We also tested two options for post-processing steps to perform on the output from BIANCA: threshold selection and masking.

As already demonstrated by Anbeek and colleagues (Anbeek et al., 2004), the choice of the threshold for the probability map output from the k-NN algorithm (calculated as the proportion of WMH and non-WMH in the feature space) has a large influence on the results: a higher threshold reduces false positives, but increases false negatives. Therefore, we tested several thresholds to define a voxel as WMH or not, in order to choose the option giving the best balance between false positives and false negatives (see Overlap with the manual mask and Results section for details). When changing the number of points included in the training dataset, a new threshold optimisation for each option was performed, given that changing the number of training points changes the probability.

Moreover, being interested in the identification of hyperintensities only in the white matter, we tested the efficacy of applying an exclusion mask to BIANCA's output, to remove false positives in the grey matter (cortical and subcortical). The mask was created automatically from segmented T1-weighted images (for Dataset 1) or FLAIR (Dataset 2) using FSL-FAST. While other approaches for this type of post-processing rely on the segmentation of white matter (WM), grey matter (GM) and cerebrospinal fluid (CSF) (Damangir et al., 2012, Dyrby et al., 2008, Samaille et al., 2012), our approach is exclusively CSF-based. The rationale behind this is that the segmentation of WM and GM is affected by WMH, which are often misclassified as grey matter. Moreover, the GM/WM contrast is very low on FLAIR images and we wanted this approach to work also in absence of a T1 weighted image (as in Dataset 2). Therefore, we used FSL-FAST to obtain a two-class segmentation (CSF and WMH + GM), extracted the cortical CSF from the CSF map (as we want to retain periventricular WMH) and dilated it to include the cortical GM. A mask, including subcortical structures (thalamus and basal ganglia) and the entorhinal cortex identified on the Harvard-Oxford atlas, was registered to the single-subjects' images and added to the exclusion mask.

### BIANCA optimization

We tested the different options and evaluated BIANCA's performance on a subsample of subjects for each dataset (21 for Dataset 1 and 109 for Dataset 2), for which a manually segmented WMH mask was available.

The algorithm optimization was performed in two phases. During the first phase we tested one or two options at a time, starting from a default sets of options (FLAIR + T1 registered in T1 space, sw = 1, no patch, any WMH load training subjects, any location for non-WMH training points, FE number of training points, threshold = 0.95, exclusion mask applied). The other options were kept constant in order to isolate the effect of each single option on the performance. In particular, on Dataset 1, we ran the following tests:

A) Multiple MRI modalities and exclusion mask (using images registered in T1 space, sw = 1, no patch, any WMH load training subjects, any location for non-WMH training points, FE number of training points, threshold = 0.95); Values tested: FLAIR only, FLAIR + T1, FLAIR only + exclusion mask applied, FLAIR + T1 + exclusion mask applied.
B) Threshold optimisation (using FLAIR + T1, sw = 1, no patch, any WMH load training subjects, any location for non-WMH training points, FE number of training points, exclusion mask applied); Values tested: 0.8, 0.85, 0.9, 0.95, 0.99.
C) Spatial weighting (using FLAIR + T1, no patch, any WMH load training subjects, any location for non-WMH training points, FE number of training points, threshold = 0.95, exclusion mask applied); Values tested: sw = 1, sw = 5, sw = 10.
D) Patch (using FLAIR + T1, sw = 1, any WMH load training subjects, any location for non-WMH training points, FE number of training points, threshold = 0.95, exclusion mask applied); Values tested: none, D = 3, D = 6, D = 9.
E) Subjects included in the training set (using FLAIR + T1, sw = 1, no patch, any location for non-WMH training points, FE number of training points, threshold = 0.95, exclusion mask applied). Values tested: 21 subjects with no restriction on WMH load, 11 (any WMH load) subjects with high WMH load or 10 subjects with low WMH load.
F) Location of non-WMH training points (using FLAIR + T1, sw = 1, no patch, any WMH load training subjects, FE number of training points, threshold 0.95, exclusion mask applied); Values tested: any, no border, surround.
G) Number (and location) of training points (using FLAIR + T1, sw = 1, no patch, any WMH load training subjects, threshold optimised for each option, exclusion mask applied); Values tested (also based on results from point F): FE + any, FE + no border, AE + any, AE + no border, FU + any, FU + no border.
H) Combination of best options (A–G).

This process led to 19 different configurations tested on Dataset 1, plus 6 threshold optimisations. In the second phase, we started from the combination of the best values for each option found in the first phase and repeated the process, to test if this was in fact an optimal solution. We also tested a subset of options in FLAIR space, to assess the influence of the reference space on the performance. Details of the second phase optimisation and the choice of the reference space are described in the supplementary material. A similar optimisation approach was adopted for Dataset 2, although we tested a subset of options on the basis of the results obtained in the first dataset and given the availability of FLAIR images only.

#### Overlap with the manual mask

To evaluate the degree of overlap between the BIANCA output and the manual mask, the following measures were calculated for each option and each subject:

• *Dice Similarity Index* (*SI*): calculated as 2 ∗ (true positive WMH voxels) / (true WMH voxels + positive voxels).
• *Voxel-level false positive ratio* (*FPR*)^1^: number of voxels incorrectly labelled as WMH (false positive, FP) divided by the total number of positive WMH voxels (i.e. voxels labelled as WMH by BIANCA).
• *Voxel-level false negative ratio* (*FNR*): number of voxels incorrectly labelled as non-WMH (false negative, FN) divided by the total number of true WMH voxels (i.e. voxels labelled as WMH in the manual mask).
• *Cluster-level FPR*: number of clusters incorrectly labelled as WMH (FP clusters) divided by the total number of positive WMH clusters (i.e. clusters labelled as WMH by BIANCA).
• *Cluster-level FNR*: number of clusters incorrectly labelled as non-WMH (FN clusters) divided by the total number of true WMH clusters (i.e. clusters labelled as WMH in the manual mask).
• *Detection error rate* (*DER*) (Steenwijk et al., 2013, Wack et al., 2012): the detection error is the sum of voxels (WMH volume) belonging to FP or FN clusters. The DER is obtained by dividing the detection error by the mean total area, calculated as the average total WMH volume by the manual mask and BIANCA output.
• *Outline error rate* (*OER*) (Steenwijk et al., 2013, Wack et al., 2012): the outline error is the sum of voxels belonging to true positive clusters (WMH clusters detected by both manual and automated segmentation), excluding the overlapping voxels. The OER is obtained by dividing the outline error by the mean total area, calculated as the average total WMH volume by the manual mask and BIANCA output.

All the measures of overlap were calculated in the reference space (i.e. T1w for Dataset 1 and FLAIR for Dataset 2). The SI was considered the overlap measure with the highest importance for the final decision, being a summary measure of overlap. Between FNR and FPR measures, we gave higher importance to having a low cluster-level FNR, as we are more interested in achieving high sensitivity to lesion detection.

#### Volumetric agreement

The volumetric correspondence between BIANCA output and manual segmentation was measured using the intra class correlation coefficient (ICC; two-way mixed model with absolute agreement definition) for the total WMH volume. This was considered the volumetric measure with the highest importance for the final decision.

On the best set of options, we also calculated the correlation between the WMH volumes extracted with BIANCA and from the manual masks, and between the WMH volumes extracted with BIANCA and the visual ratings. The rationale behind this was that if BIANCA is a valid alternative to manual segmentation, the correlation between the volumes extracted with BIANCA and the visual ratings should be as good as the correlation between the volumes extracted from the manual masks (the gold standard for WMH segmentation algorithms) and the visual ratings. To be able to correlate WMH volumes with non-volume measures (visual ratings and later age), we adjusted the WMH volume for the total intracranial volume. This was calculated as volume of the brain-extracted images (using FSL BET) from T1 images for Dataset 1 and FLAIR images for Dataset 2. Volumes expressed as a percentage of the total intracranial volume (WMHr) were correlated with the visual ratings, using Spearman's correlation.

### BIANCA validation

Once optimised, BIANCA was used to segment WMH on the full sample of Dataset 1 (85 subjects) and Dataset 2 (474 subjects). To clinically validate our tool we verified that the volumes correlated with the visual ratings (as for the algorithm optimization), but also correlated with age, which is considered a good external standard (Tiehuis et al., 2008, van den Heuvel et al., 2006). In fact, age has been related to the presence of WMHs in the literature (Gupta et al., 2015, Simoni et al., 2012), therefore a better performance of a method for assessing WMHs (either visual ratings or volumetric measurement) would presumably translate well into a closer association with age. Volumes extracted with BIANCA (using the optimised configuration found in the previous step), expressed as a percentage of the total intracranial volume (WMHr), were log transformed due to their skewed distribution (Jeerakathil et al., 2004), and correlated with Fazekas score, ARWMC score and age using Spearman's correlation.

### Reproducibility test

We tested the reproducibility of BIANCA output on a subsample of 20 subjects from Dataset 2 that have been scanned twice (mean age 68 ± 13 years, F:M = 9:11; see Test datasets for other details). The reproducibility was assessed comparing the WMH volume using a scatter plot, calculating the correlation and the ICC between the WMH volumes obtained with the two measurements, using a Bland-Altman plot (Bland and Altman, 1986, Bland and Altman, 2003) and calculating the percentage error in the volume estimation as the absolute difference between the two scans divided by their mean.

### Comparison with existing approaches

Finally, we compared the performance of BIANCA with respect to other existing approaches. Because most of the algorithms are not publicly available or easily accessible, we first performed an indirect comparison, in which we compared the performance of BIANCA in terms of SI (both total and divided with respect to WMH load) and ICC with respect to the studies reviewed by Caligiuri et al., (Caligiuri et al., 2015) dealing with WMH, or using a similar approach (kNN), but in different applications (mainly MS lesions).

We then performed a direct comparison on Dataset 1 between BIANCA and three freely available algorithms: CASCADE (Damangir et al., 2012)(ki.se/en/nvs/cascade), and the toolbox “LST: Lesion Segmentation Tool” (http://www.applied-statistics.de/lst.html) (Schmidt et al., 2012), using its two available variants: LGA (lesion growth algorithm) and LPA (lesion prediction algorithm). The details of the algorithms and their application to our data are described in the supplementary material. After finding the optimal threshold for CASCADE, and the optimal initial threshold (kappa) value for LGA, we compared the performance of the three algorithms (in terms of overlap and volumetric agreement with the manual masks) against the optimal results from BIANCA.

## Results

### BIANCA optimization

**Fig. 2:**
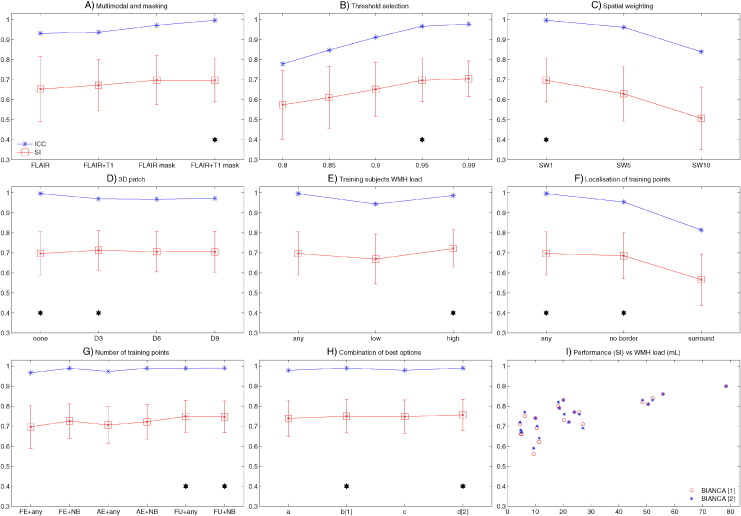
BIANCA optimisation phase I. The plots show the values of the main metrics used to evaluate BIANCA performance using different values (x axis) for the different options (panels A-H. Please refer to the main text and supplementary table S1 for details about the options). The similarity index (SI) is shown in red, with mean value (square marker) and standard deviation (error bars) across subjects. The intra class correlation coefficient (ICC) between the total WMH volume from BIANCA output and manual segmentation is shown in blue. The black stars indicate the value(s) chosen for a specific option. Panel I shows BIANCA performance (SI) for each subject against the WMH load (WMH volume in mL extracted from the manual masks. Legend: FE = Fixed Equal, AE = All Equal, FU = Fixed Unbalanced number of training points; NB = no border. Panel H legend: a = high WMH load training subjects, FU training points, no patch, any location for non-WMH training points, threshold = 0.9, mask applied; b = high WMH load training subjects, FU training points, no patch, NB location for non-WMH training points, threshold = 0.9, mask applied; c = high WMH load training subjects, FU training points, patch D = 3, any location for non-WMH training points, threshold = 0.9, mask applied; d = high WMH load training subjects, FU training points, patch D = 3, NB location for non-WMH training points, threshold = 0.9, mask applied. [1] and [2] = best options chosen.

**Fig. 3:**
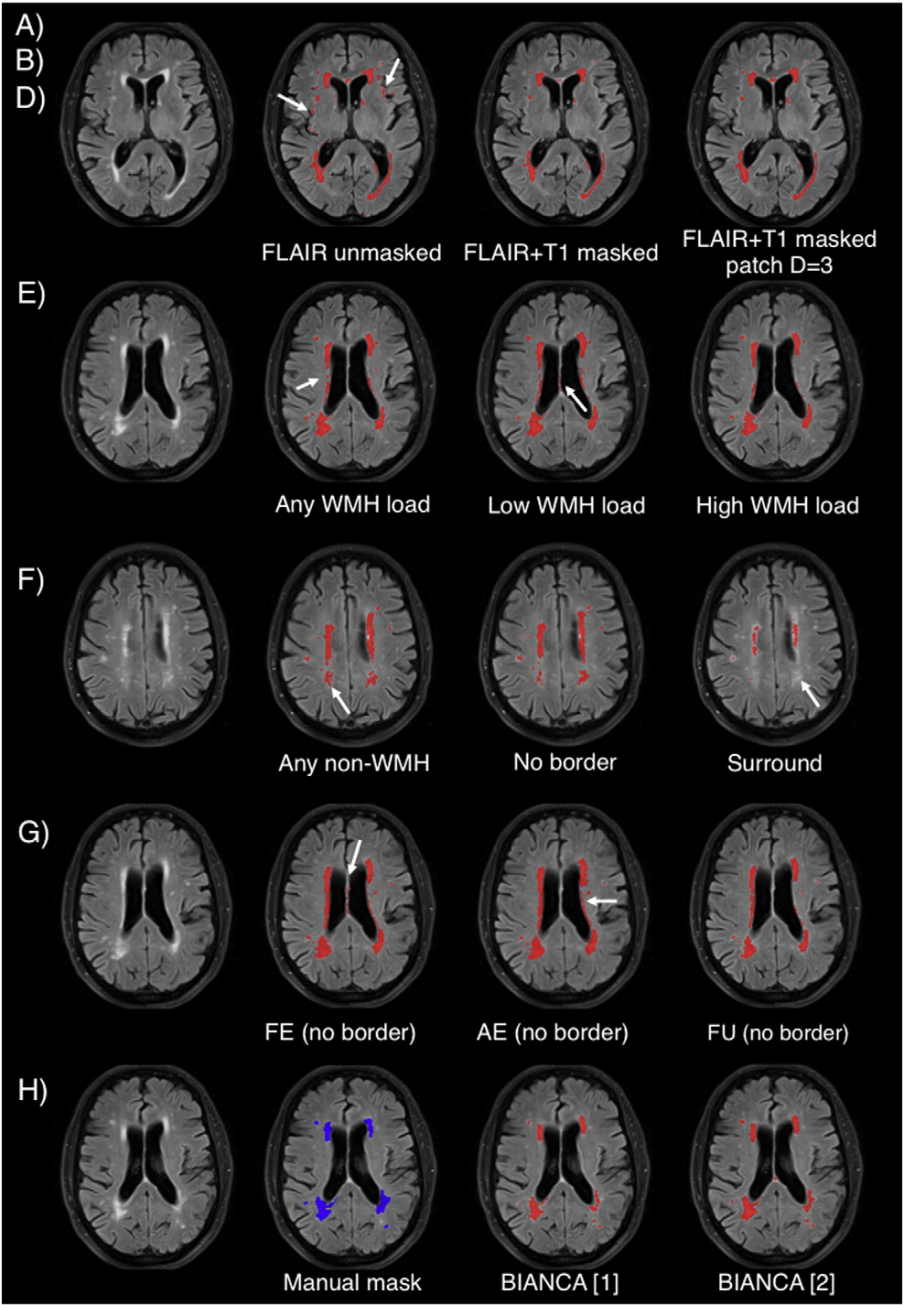
BIANCA optimisation. Examples of BIANCA output from some of the options tested (especially those not already evaluated in literature) on one subject from Dataset 1 (male, 76 years): A) and B) Multimodal MRI and masking; D) “patch” option; E) training subjects; F) localisation of training points; G) number of training points; H) combination of best options, shown next to the manual mask. The white arrows point at segmentation errors. Please refer to main text for details about the options.

Fig. 2 shows the SI (red) and ICC (blue) for the different tests relative to Dataset 1 (21 manually segmented subjects) highlighting the value chosen for each option (black star). The values of all the measures of overlap and volumetric agreement are reported in Supplementary Table S1. Fig. 3 shows examples of output from some of the options tested, especially those not already evaluated in literature.

**Fig. S2:**
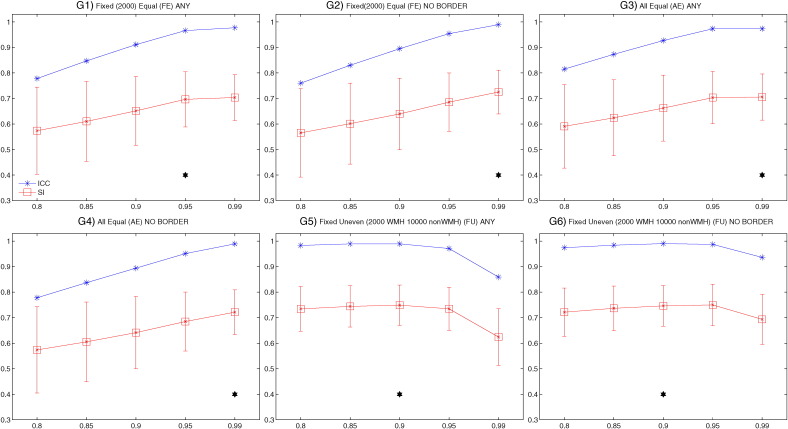
BIANCA optimisation phase I (Dataset 1) – threshold optimisations (option G in the main text). The plots show the values of the main metrics used to evaluate BIANCA performance using different values (x axis) for the different options (please refer to the main text and supplementary table S1 for details about the options). The similarity index (SI) is shown in red, with mean value (square marker) and standard deviation (error bars) across subjects. The intra class correlation coefficient (ICC) between the total WMH volume from BIANCA output and manual segmentation is shown in blue. The black stars indicate the value(s) chosen for a specific option.

In particular, we observed that:

A) The use of an exclusion mask always improved the performance (higher SI and ICC). On unmasked images, use of intensity information from T1 images increased the accuracy of the segmentation. On masked images the SI from FLAIR only or FLAIR + T1 were very similar, but the ICC was higher using both modalities (Fig. 2.A).
B) The best thresholds were 0.95 and 0.99. They had similar SI and ICC, but the former had lower FNR cluster, so it was selected as best threshold (Fig. 2.B).
C) The spatial weighting giving the best results was sw = 1 (Fig. 2.C).
D) Similar results were obtained when not using any additional local average intensity features (no patch) or including the average intensity using a 3D patch of D = 3 (Fig. 2.D).
E) The highest SI was obtained when using the 11 subjects with high WMH load. Similar SI and higher ICC were obtained using all 21 subjects (any WMH load) (Fig. 2.E). As the number of subjects used for the 3 options was not the same (21 for any WMH load, 10 low WMH load and 11 for high WMH load), we ran an additional test using 10 subjects with any WMH load, to have a comparable number of training subjects with the other two options. The performance using 10 subjects with any WMH load was similar to using 21 subjects and lower than using 11 subjects with high WMH load (results not shown).
F) Preferentially using non-WMH points at the edge of the WMH masks (surround option) gave the worst results. Similar results were obtained using non-WMH points from anywhere outside the mask (any option) or excluding the points close to the edge of the WMH masks (no border option) (Fig. 2.F).
G) During threshold optimisation for each option, best results were obtained with threshold 0.95 and 0.99 for the FE and AE options and with threshold 0.85 and 0.9 when using the FU option (see Supplementary Table S1, and Supplementary Fig. S2). When comparing the options (Fig. 2.G), we used the higher threshold for all of them. Among the optimised options, the use of a different number of training points for WMH (2000) and non-WMH (10,000) gave the best results (even when comparing them using the lower optimal threshold in the threshold optimisation phase).
H) Combining the results obtained with the previous tests (Fig. 2.H), the best results in terms of highest SI and lowest cluster-level false positive ratio were obtained using: FLAIR + T1 images, threshold = 0.9, exclusion mask, sw = 1, no patch (BIANCA[1]) or 3D patch D = 3 (BIANCA[2]), high WMH load training subjects, no border option, different number of training points for WMH (2000) and non-WMH (10,000). Results of all the metrics for the optimised settings are reported in Table 1.

Fig. 2.I plots BIANCA performance (SI using the best options) versus WMH load (mL) (calculated from the manual mask in the reference space) and shows that the performance is higher for subjects with higher WMH load.

**Fig. S3:**
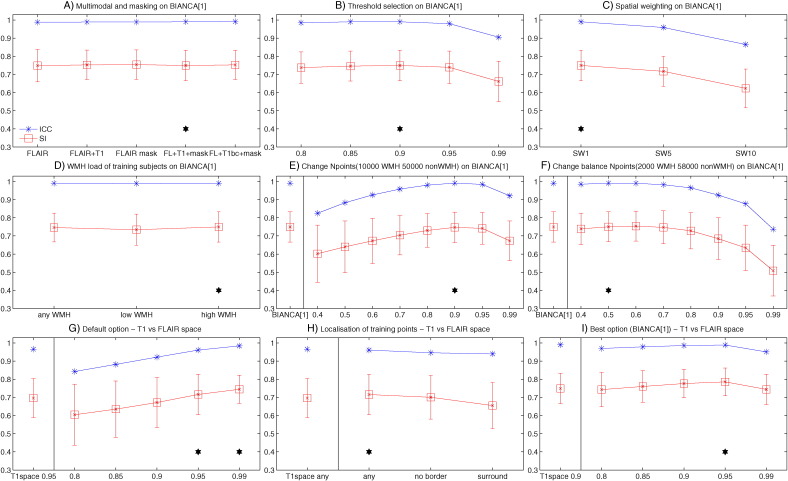
BIANCA optimisation phase II (A-F) and choice of reference modality (G-I) (Dataset 1). The plots show the values of the main metrics used to evaluate BIANCA performance using different values (x axis) for the different options (please refer to the supplementary material and supplementary Table S2 for details about the options). The similarity index (SI) is shown in red, with mean value (square marker) and standard deviation (error bars) across subjects. The intra class correlation coefficient (ICC) between the total WMH volume from BIANCA output and manual segmentation is shown in blue. The black stars indicate the optimal alternative options for the specific optimisation step.

The second optimisation phase, performed on BIANCA[1], confirmed that the chosen values for each option were still an optimal solution (giving comparable or higher performance to the tested alternatives) when varying them, and the use of FLAIR as reference space gave similar results. Details of these analyses and results are reported in the supplementary material, Supplementary Fig. S3, and Supplementary Table S2.

The reported processing time for WMH segmentation on a 2.93 GHz Intel Xeon CPU for one subject in Dataset 1 was approximately 2 min with option BIANCA[1] and 3 min with BIANCA[2]. For the post-processing step of generation of the exclusion mask, the reported processing time was approximately 10 min.

**Fig. S4:**
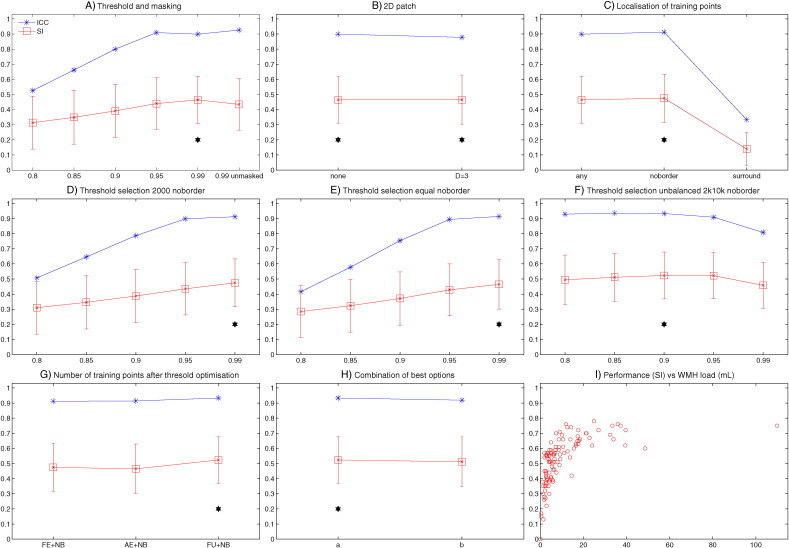
BIANCA optimisation on Dataset 2. The plots show the values of the main metrics used to evaluate BIANCA performance using different values (x axis) for the different options (panels A-H. Please refer to the main text and supplementary Table S3 for details about the options). The similarity index (SI) is shown in red, with mean value (square marker) and standard deviation (error bars) across subjects. The intra class correlation coefficient (ICC) between the total WMH volume from BIANCA output and manual segmentation is shown in blue. The black stars indicate the value(s) chosen for a specific option. Panel I shows BIANCA performance (SI) for each subject against the WMH load (WMH volume in mL extracted from the manual masks. Legend: FE = Fixed Equal, AE = All Equal, FU = Fixed Unbalanced number of training points; NB = no border. Panel H legend: a = high WMH load training subjects, FU training points, no patch, no border location for non-WMH training points, mask applied, threshold 0.9; b = high WMH load training subjects, FU training points, 2D patch D = 3, no border location for non-WMH training points, mask applied, threshold 0.9.

A similar approach was used to evaluate the performance of BIANCA on Dataset 2 (on 109 manually segmented subjects), and the relative results are reported in Supplementary Fig. S4 and Supplementary Table S3. In this case a subset of options was tested, on the basis of the results obtained in the first dataset (spatial weighting = 1, 20 subjects with high WMH load included, patch D = 3 and no patch only), and also because for Dataset 2 only FLAIR images were available (no multimodal option). The analyses led to the choice of similar settings: threshold = 0.9, exclusion mask, sw = 1, no patch, high WMH load training subjects, no border option, unbalanced number of training points for WMH and non-WMH classes (FU option). Results for the optimised settings are reported in Table 1.

In this dataset we had excluded subjects with chronic, sub-acute or acute infarcts larger than 2 cm or other major brain alterations. However, to further ensure that the lower performance of BIANCA on Dataset 2 was due to the image quality rather than the presence of vascular damage, we evaluated BIANCA performance on a subsample of 82 subjects (out of 109) that did not have any lacunar small infarcts visible as restricted diffusion on DWI images (exclusion of DWI positive scans). The results with the optimal option were very similar to the ones obtained on the original sample: SI = 0.50, ICC = 0.921.

The reported processing time for WMH segmentation on a 2.93 GHz Intel Xeon CPU for one subject in Dataset 2 with the optimised setting was < 2 min (approximately 110 s). For the post-processing step of generation of the exclusion mask, the reported processing time was approximately 10 min.

The correlations between BIANCA volumes, manual volumes and visual ratings are reported in Table 1. In Dataset 1, either of the two best options for BIANCA gave correlations of the WMHr with the visual ratings that were higher than those between the WMHr derived from the manual masks and the visual ratings, although they were not significantly different when testing the equality of the two correlation coefficients, with the two correlations sharing one variable in common (Lee and Preacher, 2013, Steiger, 1980): Spearman's rho_21_ BIANCA[1] WMHr – Fazekas = 0.944**, BIANCA[2] WMHr – Fazekas = 0.935**, manual WMHr – Fazekas = 0.933**; Spearman's rho_21_ BIANCA[1] WMHr – ARWMC = 0.947**, BIANCA[2] WMHr – ARWMC = 0.953**, manual WMHr – ARWMC = 0.943** (**p < 0.01). Similarly to Dataset 1, also for Dataset 2 the correlations of the WMHr (using the best options for BIANCA) with the visual ratings were higher, although not significantly different than the correlation between WMHr derived from the manual masks and the visual ratings: Spearman's rho_109_ BIANCA WMHr – Fazekas = 0.782**, manual WMHr – Fazekas = 0.742**; Spearman's rho_109_ BIANCA WMHr – ARWMC = 0.785**, manual WMHr – ARWMC = 0.746**, **p < 0.01.

### BIANCA validation

**Fig. 4:**
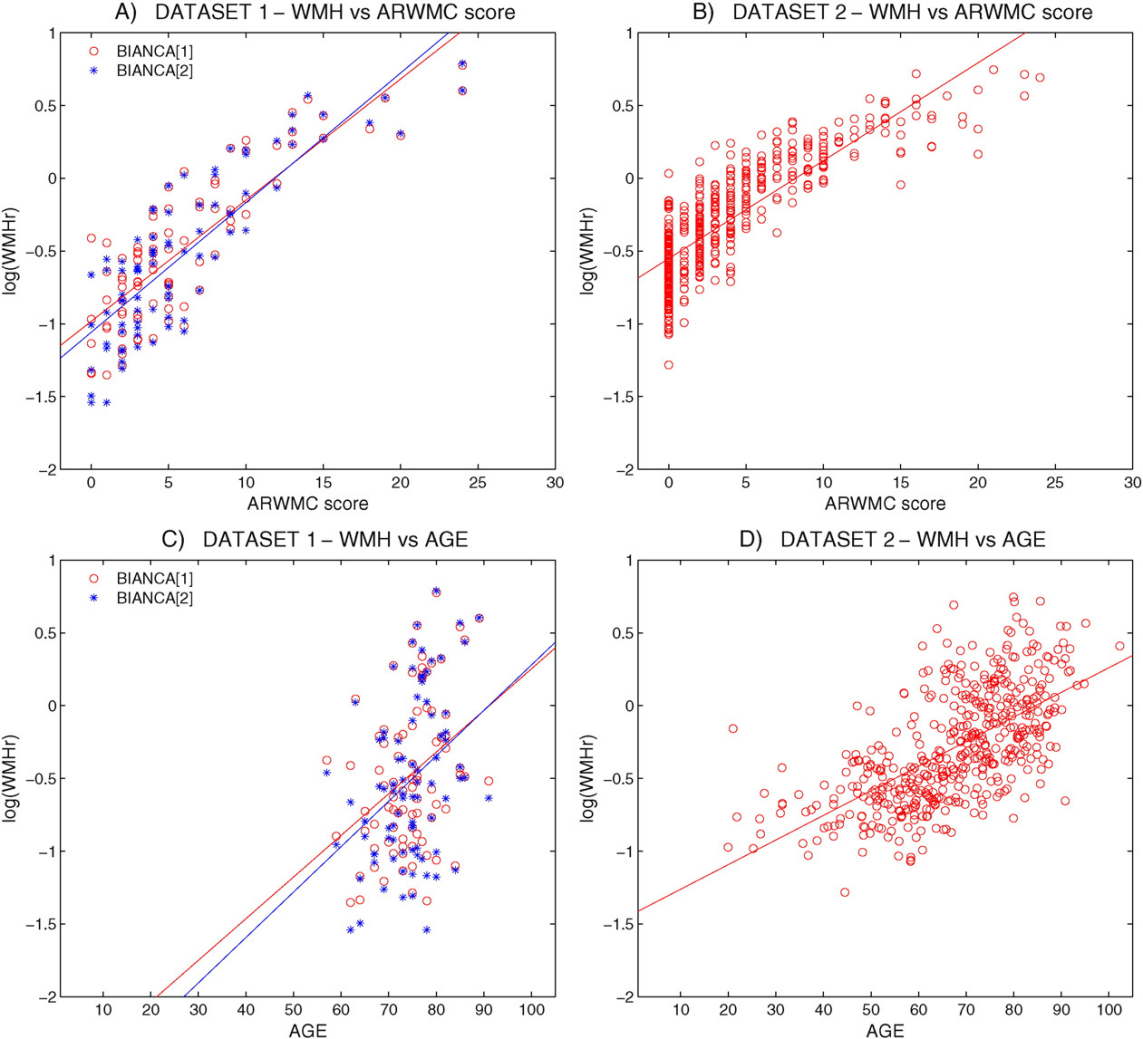
BIANCA validation. Scatter plot of the WMH distribution according to ARWMC score (A and B) and age (C and D). WMH volumes were extracted with BIANCA using the optimised configuration(s) for each dataset, expressed as a percentage of intracranial volume, log transformed and plotted against ARWMC or age. A linear fitting is also shown. See main text for correlation values.

In both whole datasets (85 subjects for Dataset 1 and 474 for Dataset 2) the WMHr remained highly significantly correlated with the visual ratings (Dataset 1: Spearman's rho_85_ BIANCA[1] WMHr – Fazekas = 0.766**, BIANCA[2] WMHr – Fazekas = 0.772**, BIANCA[1] WMHr – ARWMC = 0.795**, BIANCA[2] WMHr – ARWMC = 0.817**; Dataset 2: Spearman's rho_474_ BIANCA WMHr – Fazekas = 0.838**, BIANCA WMHr – ARWMC = 0.840**, **p < 0.01). The distribution of log(WMHr) with respect to the ARWMC score is shown in Fig. 4 (panels A and B).

In both datasets the WMHr calculated with BIANCA showed a significant correlation with age (Dataset 1: Spearman's rho_85_ BIANCA[1] WMHr – age = 0.367**, BIANCA[2] WMHr – age = 0.371**; Dataset 2: Spearman's rho_474_ BIANCA WMHr – age = 0.659**, **p < 0.01), and the distribution of log(WMHr) with respect to age (see Fig. 4, panels C and D) shows a linear trend.

When comparing the results obtained with BIANCA with visual ratings, in Dataset 1 the correlation of the WMHr with age was higher, although not significantly different, than the correlation between the visual ratings and age (Spearman's rho_85_ BIANCA[1] WMHr – age = 0.367**, BIANCA[2] WMHr – age = 0.371**, Fazekas - age = 0.352**, ARWMC - age = 0.326**, **p < 0.01). In Dataset 2, the correlation of the WMHr with age was significantly higher (Lee and Preacher, 2013, Steiger, 1980), than the correlation between the visual ratings and age: Spearman's rho_474_ BIANCA WMHr – age = 0.659**, Fazekas - age = 0.574**, **p < 0.01 (test of the equality of correlation coefficients with one variable in common: z-value 4.259, *p* < 0.01), ARWMC - age = 0.589**, **p < 0.01 (test of the equality of correlation coefficients with one variable in common: z-value 3.549, *p* < 0.01).

### Reproducibility test

**Fig. 5:**
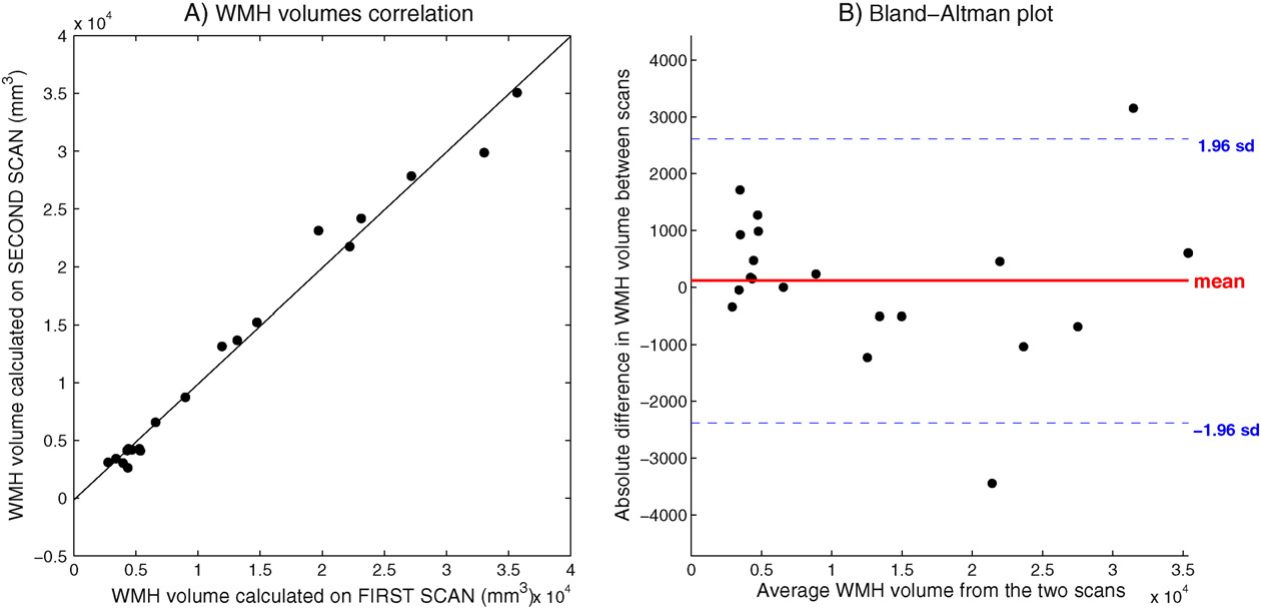
Reproducibility test. Scatter plot (A) and Bland-Altman plot (B) of the WMH volumes calculated on the images obtained from 20 subject (Dataset 2) with the same scanner and protocol at different times (within-scanner reproducibility).

The results of the reproducibility test on the 20 subjects from Dataset 2 are shown in Fig. 5. The two measurements of WMH volumes were significantly correlated (Spearman's rho = 0.961, *p* < 0.001), their volumetric agreement was ICC = 0.996, and the average percentage error between the WMH volumes was 10.53 ± 12.22%.

### Comparison with existing approaches

The results of the comparisons are reported in Table 2. From the indirect comparison it can be observed that the average SI and ICC values obtained by BIANCA are in line with the values reported in previous studies, with a higher performance when subjects have higher WMH load.

**Fig. S5:**
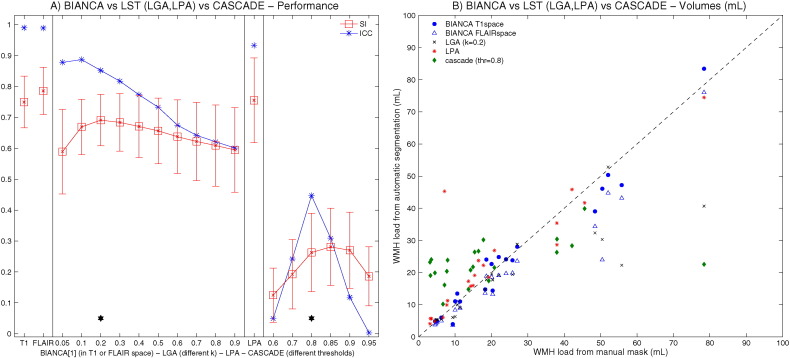
Direct comparison with available tools. Panel A shows the similarity index (SI, red), with mean value (square marker) and standard deviation (error bars) across subjects and the intra class correlation coefficient (ICC, blue) between the total WMH volume from BIANCA (in T1w or in FLAIR space), CASCADE (for different thresholds), LGA (for different kappa values, threshold 0.5) and LPA (threshold 0.5) and manual segmentation. The black star indicates the optimal options for CASCADE and LGA that were chosen for comparison against BIANCA. Panel B shows volumes calculated from BIANCA, CASCADE, LGA and LPA for each subject against the WMH load (WMH volume in mL extracted from the manual masks in the reference space used by the algorithm).

**Fig. S6:**
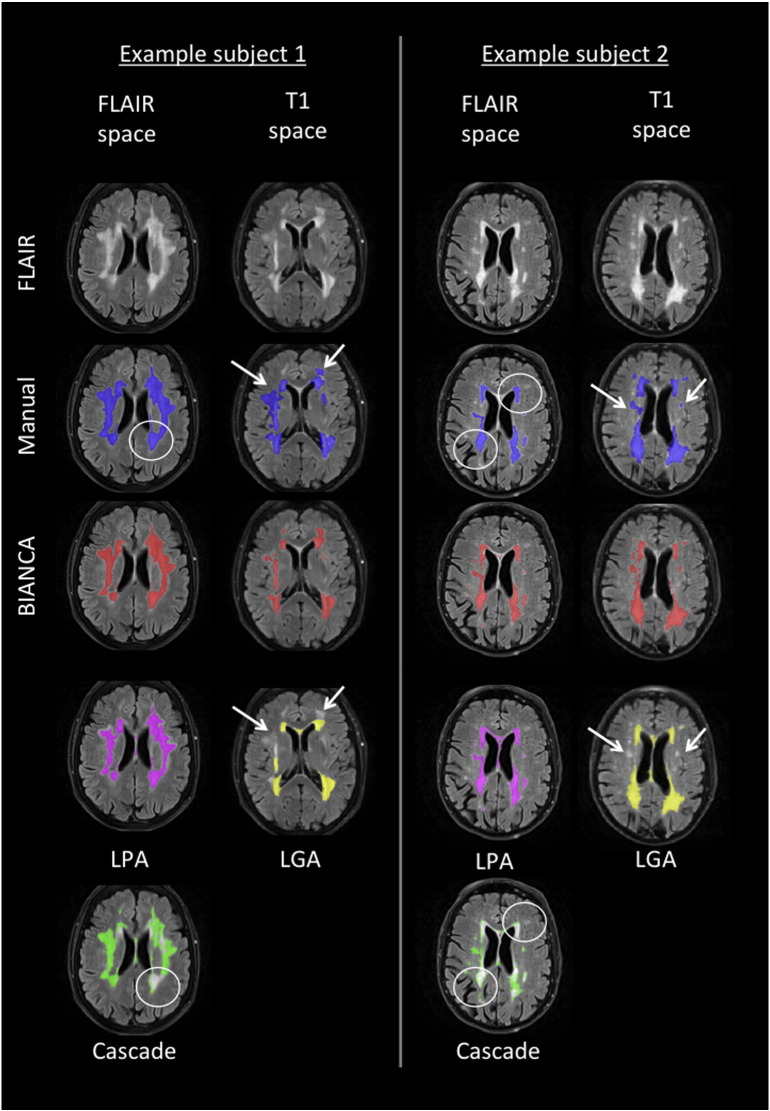
Example WMH segmentation with different tools. FLAIR images from two subjects are shown, together with the manual mask and the output from the different segmentation tools either in FLAIR space or T1w space, according to the reference space used for WMH segmentation by the algorithm. CASCADE (threshold = 0.8) and LPA gave the best performance on subject number one, while subject number 2 was the one giving the best LGA (kappa = 0.2) performance. White arrows and circles highlight the main segmentation errors by CASCADE and LGA, while BIANCA and LPA show good performance in both examples.

The direct comparison between BIANCA, LGA (Schmidt et al., 2012), LPA and CASCADE (Damangir et al., 2012) on Dataset 1 showed that BIANCA performance was higher than CASCADE and LGA and comparable to LPA both in terms of overlap (BIANCA SI = 0.75, LGA SI = 0.69; LPA SI = 0.76, CASCADE SI = 0.26) and of volumetric agreement (BIANCA ICC = 0.990, LGA ICC = 0.852; LPA ICC = 0.933, CASCADE ICC = 0.447) with the manual masks. Details are shown in Fig. S5, Fig. S6 and Supplementary Table S4.

## Discussion and conclusion

We present BIANCA, a new automated algorithm for the segmentation of white matter hyperintensities of presumed vascular origin that we optimised and validated on two different datasets representative of clinical populations in whom the clinical importance of WMH is recognized: a “predominantly neurodegenerative” cohort including people with, or at risk of, AD and a “predominantly vascular” cohort including people with, or at risk of, vascular cognitive impairment.

The two datasets had different MRI protocols and included both research sequences as well as diagnostic standard MRI sequences commonly used in routine clinical practice.

The performance was evaluated by means of comparison with manually segmented WMH masks in terms of overlap and volumetric agreement. The optimal configuration used intensity features from FLAIR and T1-weighted images with no local averaging (or only a small amount) and normalised MNI spatial coordinates. The best options for the training dataset were the use of voxels from subjects with high WMH load, a different number of training points for WMH and non-WMH classes, and avoiding using the voxels near the lesions' edges as non-WMH training points (the “no border” option). The results were further improved in the post-processing step by using a threshold of 0.9 and applying an exclusion mask for grey matter and subcortical structures.

As shown in previous studies (Anbeek et al., 2004, Steenwijk et al., 2013) the use of more than one MRI modality increased the accuracy of the classification. However, BIANCA is very flexible, as it can use as many modalities as available, or useful, for a specific dataset, showing good performance both with FLAIR only and with FLAIR plus T1. Other images, not tested in this study, can also be included (e.g. like proton density, diffusion, or tissue priors). All the modalities need to be registered to a consistent reference MRI modality, but BIANCA is flexible in terms of which modality to use as reference, unlike other available tools, which have a predefined reference space (FLAIR for CASCADE, T1w for LGA). In this study we decided to use T1 images as reference for the main analyses on Dataset 1, to avoid down-sampling it when registered to FLAIR space. However, we repeated the analyses with a sub-set of options also on data registered in FLAIR space (which has the greatest intensity contrast for WMH) and obtained very similar results (see Supplementary material), suggesting that the choice of the reference modality does not have a big impact on the results and the user can choose the reference modality depending on the aim of the study and on the resolution of the data.

As demonstrated in previous studies (Anbeek et al., 2004), information about the coordinates of a voxel was useful, as in some regions of the brain WMH are more likely to occur than in others. From our tests, there was no need of more focal weighting for spatial coordinates (optimal sw = 1). A higher sw would probably be more beneficial in populations with a more specific spatial location of the lesions.

BIANCA also offers the possibility to include additional intensity features, calculating a local average within a kernel (patch) of size D. The inclusion of intensity information about a small neighbourhood of each voxel has been proposed before (Dyrby et al., 2008, Lao et al., 2008), but not tested in a kNN-based algorithm. With BIANCA, the patch can be applied in 3D or 2D, with the latter option being useful in case of highly anisotropic voxels, like in our second dataset. We obtained the best results with no patch or local averaging within a small kernel, however, we did not test the use of multiple patch sizes, an option that is also available with BIANCA.

Regarding the selection of subjects to be included in the training dataset, we reached a good performance by using only 10 subjects for Dataset 1 and 20 subjects for Dataset 2, less than or in line with previous studies (Anbeek et al., 2004, Steenwijk et al., 2013). In particular, the best results were obtained when using images from subjects with high WMH load, probably because the features, especially intensities, of the WMH were less ambiguous and the number of WMH voxels was more plentiful. This result is an advantage during the training phase as subjects with high WMH load are also easier to manually segment. Furthermore, a better performance was obtained using only 11 training subjects with high WMH load (in Dataset 1) versus using 21 subjects with varying WMH loads (and also using the same number of subjects with varying WMH loads, results not shown).

Regarding the number of voxels included in the training dataset, in the algorithm proposed by Anbeek and colleagues (Anbeek et al., 2004) a fixed number (20%) of the training voxels was randomly selected for inclusion in the learning set. With BIANCA a substantial improvement in the segmentation's accuracy was achieved by introducing the possibility to change the number of training voxels and use an unbalanced number of samples from the two classes (FU option). This is probably due to the fact that non-WMH voxels are more heterogeneous, as they can belong to any tissue type. Therefore, using more non-WMH compared to WMH voxels gives a better representation of the characteristics of the non-WMH class. In this study we did not change the number of training points for the FE and FU options (2000 voxels for the WMH class for each subject included in the training dataset, and 2000 or 10,000 for the non-WMH class for the FE and FU options respectively), focusing on testing the impact of the novel option of using an unbalanced number of points for the two classes. Given the increased performance using this option, we also tested the effect of increasing the total number of points either maintaining the same ratio between the two classes (1:5) or increasing only the number of training points for the non-WMH class (up to 1:29 ratio) (see Supplementary material for details). In both cases, after threshold optimisation, the results showed similar performance to the optimal settings found with lower number of training points. Although further tests using different combinations of number of points and ratio between the two classes could be performed as part of the optimisation phases, these results suggest that the BIANCA segmentation is already accurate when using a modest number of training points and does not benefit much from increasing this number.

Another important improvement in the algorithm was the possibility to choose the location of non-WMH training voxels. In fact, using the non-WMH voxels near to the lesion's edge was observed to cause a decrease in the performance (“surround” option), while excluding them was found to be beneficial (“no border” option). This is not surprising, as manual segmentation is variable within and between operators (Mantyla et al., 1997), especially at the edge of WMHs, as they typically don't have sharp boundaries. In our study, this could also be due to the fact that FLAIR images were registered to T1, possibly introducing interpolation errors at the lesion's edge. However, the surround option gave the worst performance also on Dataset 2 (on FLAIR images only) and when repeating the analyses on Dataset 1 in FLAIR space (see supplementary material for details), suggesting that avoiding using voxels at the lesions' edges as training voxels is generally beneficial.

In the post-processing step, a threshold of 0.9 was found to be optimal for both datasets tested in this study. This is quite different from other studies (Anbeek et al., 2004, Steenwijk et al., 2013), but it needs to be kept in mind that the threshold depends on the number of nearest neighbours used in the algorithm (k) and on the number of training points used. Also, an advantage of the k-NN approach (Anbeek et al., 2004) is that obtaining a WMH probability map rather than a binary map allows the threshold to be changed depending on the purpose of the segmentation. In this study, we wanted to minimize false negatives, but the user can decide on their own threshold based on the acceptable ratio between false positives and false negatives for a specific study.

Finally, the use of a mask (automatically generated) excluding grey matter, cerebellum and subcortical structures was found to be an effective method for removing false positives, as typically FLAIR images present hyperintensities in cortical areas and flow artefacts in and around the 4th ventricle. The novel CSF-based masking used here crucially enabled us to work with the highly anisotropic voxels of the Dataset 2, which is not unusual in clinical imaging in our experience, and in absence of a T1 weighted image.

The fact that we obtained more than one optimal settings of options/parameters (both in the first and second optimisation phase) is due to the great variation in image acquisitions and the complexity of lesion segmentation and its intended use. We believe that the parameters should be adapted to each dataset, either using a quantitative method like the two-phase optimisation procedure above or by careful qualitative assessment. Due to the large range of types of image acquisitions that are employed in clinical practice and research studies it is highly unlikely that a single set of parameters will give a good performance over all datasets and therefore we explicitly aim to optimise these for each dataset.

For any one particular dataset, although the primary metrics used to evaluate BIANCA performance (SI and ICC) showed good agreement (i.e. usually the best option was the one with higher SI and ICC), this was not always the case, as more than one set of options can give the best performance according to different metrics. Moreover, the results are always a balance between FPR and FNR. In this study we gave higher importance to having a low cluster-level FNR, as we are more interested in achieving high sensitivity to lesion detection, but the user can decide which metric(s) to prioritise to select the best set of options for a specific study.

With the optimised settings described before, BIANCA showed an average SI of 0.76, which is regarded as very good (Anbeek et al., 2004, Bartko, 1991, Caligiuri et al., 2015) and is in line with previous studies using kNN (Anbeek et al., 2004, Steenwijk et al., 2013) and other methods (see Table 2). Also, as shown in Fig. 2.I., and Fig. S4.I, the higher performance (SI) achieved in subjects with higher WMH load is in line with the literature (see Table 2 and (Dyrby et al., 2008, Wack et al., 2012)). The comparison of different methods is not straightforward, because it depends on the pulse-sequence, the reference segmentation, the pathology, the heterogeneity of the sample and the lesion burden (Steenwijk et al., 2013). For this reason we also directly tested three freely available tools (CASCADE, (Damangir et al., 2012), LGA (Schmidt et al., 2012) and LPA) on our data. The results showed that BIANCA outperformed CASCADE and LGA both in terms of overlap and volumetric agreement with the manual masks, while showed comparable performance with respect to LPA (see Table 2, Supplementary Table S4, Fig. S5, Fig. S6). CASCADE gave the worst performance on our dataset, but the substantial amount of the false positive WMH were localised in the cortex and in the subcortical structures, which in our approach are masked out with the automatic exclusion mask. In fact, when applying our exclusion mask to the output from CASCADE, we observed an increase in the performance (SI from 0.26 to 0.33 and ICC from 0.447 to 0.633, see Supplementary Table S4). Although a comprehensive comparison with other methods would require a separate study, these results suggest that BIANCA is a promising and competitive tool for WMH segmentation.

In the second dataset, the average SI was around 0.52, probably mainly due to the use of non-isotropic FLAIR images, but also to the heterogeneity in the sample in terms of pathology and WMH load (101 subjects from a population-based study, with age range 20–102 years, ARWMC score range 0–24). Although further evaluations are undoubtedly needed, the additional results on the subset of subjects that did not have any lacunar small infarcts visible as restricted diffusion on DWI images (DWI positive), suggest that the performance of BIANCA is more related to the quality of the data than to clinical features of the cohort.

The correlations of the WMHr with the visual ratings were slightly higher, than the correlation between WMHr derived from the manual masks and the visual ratings, making BIANCA an acceptable substitute for the manual segmentation of WMHs.

The second aim of our study was to validate BIANCA on two clinical cohorts of patients. In both datasets the WMHr were highly correlated with the visual ratings. Moreover, the correlations of the WMHr with age were comparable or significantly higher than those between the visual ratings and age, showing that BIANCA is also a good substitute for qualitative evaluation of WMHs, which are still frequently used but are time consuming and operator-dependent.

We tested the reproducibility of BIANCA within-scanner, to further evaluate the robustness of our method. We obtained good agreement between the volumes extracted from the two scans (Spearman's rho = 0.961, ICC = 0.996), suggesting that BIANCA can be a promising tool for further applications on more datasets.

The primary focus of the present study was to optimise BIANCA for use in cross-sectional studies. In fact, there is an increasing number of important large cross-sectional homogeneous studies that urgently need an automated tool like BIANCA. For example, studies like the OXVASC study, Dataset 2 in this study (Rothwell et al., 2004), the Whitehall study (Filippini et al., 2014), and the UK Biobank study (http://imaging.ukbiobank.ac.uk), which focus on ageing and diseased populations for potential biomarkers and understanding of ageing/disease processes, would clearly benefit from an automated method for the identification and quantification of WMH. With the size of such datasets becoming larger and larger (up to 100,000 subjects for UK Biobank), it is increasingly infeasible to perform segmentation manually and this makes the development and availability of automated tools urgently needed. BIANCA is currently not optimised for application on longitudinal data. An algorithm optimised for working on longitudinal data would require a different approach, which will be the objective for future studies.

A limitation of our method is that BIANCA is not completely automatic, as it requires a training dataset of manually segmented images when applied to data from other scanners or other acquisition protocols. The manual-labelling step is time consuming and requires expertise in WMH identification, but is necessary as the characteristics of FLAIR and T1 images varies among scanners and pulse-sequences. However, we demonstrated that we were able to reach good performance with only 10–20 subjects. This could still represent a disadvantage for small studies, but a relatively negligible effort in large cross-sectional studies with hundreds of subjects, as the ones mentioned above. We showed that higher accuracy is achieved when using subjects with high WMH load as training subjects, which are also the easiest to manually segment, as the hyperintensities are more visible. It will be important to test in future whether BIANCA can be trained on one dataset and used in another dataset acquired with the same protocol, further reducing manual intervention. This would also make BIANCA applicable to multi-centric studies.

Another limitation is the necessity to use an exclusion mask of grey matter, cerebellum and subcortical structures to decrease the amount of false positives, as typically FLAIR images present hyperintensities in cortical areas and flow artefacts around the 4th ventricle. Therefore BIANCA is currently not able to detect cortical and cerebellar abnormalities. In this study we excluded subjects with large vascular lesions or with neoplastic, developmental or inflammatory abnormalities, in order to specifically focus on white matter hyperintensities of presumed vascular origin (see Test datasets). Future studies will focus on the segmentation of other types of lesions, for example multiple sclerosis lesions.

As a limitation of this specific study, we did not optimise the value of k, but selected a value of 40 based on the literature (Steenwijk et al., 2013). However, Steenwijk and colleagues (Steenwijk et al., 2013) tested other k values (20, 80, 160) and showed that k in the current range is suitable for this type of segmentation problem, without the need to increase k and, consequently, the processing time. Moreover, we did not test all the possible configurations of the options available in BIANCA, given the very large number of possibilities. Instead, we aimed to show the effect of varying the values of the single options on the performance and to suggest a two-phase approach that can be used to optimise BIANCA for any dataset, as the flexibility of the algorithm allows the user to test any possible combination.

To conclude, in this work we optimised and validated BIANCA, an algorithm for WMHs segmentation that is:

- Fully automated, only requiring time and expertise to manually segment a small number of images, with well identifiable WMHs;
- Multimodal or capable of working with a single modality (from either 2D or 3D acquisitions);
- Flexible: allows the user to change many options, not only related to the MRI modalities;
- Generalizable to data acquired at different times from different scanners, as we tested it on two very different datasets in terms of scanner, sequences, and subjects' pathology;
- Robust: it shows good reproducibility within-scanner;
- Computationally lean (< 3 min CPU time for WMH segmentation);
- Competitive with respect to existing methods: it showed similar or higher performance compared to other approaches already proposed in the literature.
- Freely available soon to be released (beta version) as part of FSL (FMRIB software FSL)

Our results show that the measure of WMH load (WMH volume) extracted with BIANCA is a reliable substitute for manual measurements of WMH on the tested datasets. This suggests that BIANCA can be a promising tool for large cross-sectional cohort studies, and routine MR diagnostic scans, as it showed good correlation with visual ratings and a correlation with age that was comparable to or higher than visual ratings. Moreover, the availability of localization-specific measurement of WMH (WMH maps) provides the possibility to perform more detailed evaluations of WMH or to use the WMH maps as a voxel-wise nuisance variable, to disentangle the contribution of WMH from other MRI abnormalities.

The following are the supplementary data related to this article.

Supplementary material Image 1

## Funding

The Oxford Vascular Study has been funded by Wellcome Trust, Wolfson Foundation, UK Stroke Association and the NIHR Oxford Biomedical Research Centre. PMR is in receipt of an NIHR Senior Investigator Award and a Wellcome Trust Senior Investigator Award. LL has been funded by the China Scholarship Council. LG, WK and UGS are supported by the NIHR Oxford Biomedical Research Centre.

## Figures and Tables

**Table 1:** Results of BIANCA optimisation. Measures of overlap and volumetric agreement with the manual masks for the optimised settings on the two datasets are reported, together with the correlations between BIANCA volumes, manual volumes and visual ratings.

BIANCA option	Overlap with manual mask						Volumetric correspondence				
	SI	FPR	FNR	FPR clusters	FNR clusters	DER	OER	ICC	BIANCA WMH vs manual WMH⁎	WMHr vs ARWMC total⁎	WMHr vs Fazekas total⁎
BIANCA [1] dataset 1	0.75	0.22	0.26	0.77	0.02	0.03	0.47	0.990	0.961	0.947	0.944
BIANCA [2] dataset 1	0.76	0.22	0.25	0.67	0.03	0.03	0.46	0.990	0.953	0.953	0.935
BIANCA dataset 2	0.52	0.46	0.45	0.76	0.30	0.19	0.76	0.919	0.861	0.785	0.782

⁎ Spearman's correlation (all correlations were significant at *p* < 0.01).

**Table 2:** Comparison with existing approaches.

Method/Paper	Type of method^a^	Image modality	Population/Study	ICC	SI (total and for different WMH load^b^)				
					TOTAL	< 5 mL	5–10 mL	10–15 mL	> 15 mL
*Indirect comparison*									
BIANCA	S	multimodal	Neurodegenerative (option 1)	0.99	0.75	0.69	0.66	0.68	0.80
BIANCA	S	multimodal	Neurodegenerative (option 2)	0.99	0.76	0.70	0.68	0.70	0.80
BIANCA	S	multimodal	Vascular	0.93	0.52	0.41	0.53	0.63	0.68
(Steenwijk et al., 2013)	S	3DT1 and 3DFLAIR	MS	0.92	0.75	0.65	0.72	0.73	0.81
(Steenwijk et al., 2013)	S	3DT1 and 3DFLAIR	Hypertension	0.96	0.84	0.78	0.92	0.79	0.91
(Anbeek et al., 2004)	S	T1, IR, PD, T2, FLAIR	arterial vascular disease	/	0.80	0.50	0.75		0.85
(Dyrby et al., 2008)	S	T1, T2, FLAIR	elderly subjects (LADIS)	/	0.56	0.45		0.62	0.65
(Ji et al., 2013)	S	FLAIR	WM disease	/	0.87				
(Yoo et al., 2014)	S	FLAIR	Longitudinal study ageing and dementia	0.98	0.76	0.59	0.73		0.85
(Simões et al., 2013)	S	FLAIR	HC, MCI	/	0.68	0.51		0.70	
(Herskovits et al., 2008)	S	T1, T2, spin-density, FLAIR	Diabetes (ACCORD-MIND study)	/	0.60				
(Beare et al., 2009)	S	T1, T2, FLAIR	HC	0.9	0.58	0.47	0.55	0.56	
(Schmidt et al., 2012)	U	3D GRE, T1 and FLAIR	MS	/	0.75	0.67	0.76	0.82	0.85
(Khayati et al., 2008)	U	FLAIR	MS	/	0.75	0.73	0.75		0.81
(Sajja et al., 2006)	U	PD and T2-FLAIR	MS	/	0.78	0.67		0.84	
(Admiraal-Behloul et al., 2005)	U	PD, T2 and FLAIR	PROSPER - risk for/pre-exhisting vasc disease	0.98	0.75	0.70	0.75		0.82
(Jeon et al., 2011)	U		SVD	/	0.90				
(Shi et al., 2013)	U	T, FLAIR and DWI	Acute Infarction	0.99	0.84				
(Khademi et al., 2012)	U	FLAIR	subject with lesions	/	0.83				
(Gibson et al., 2010)	U	FLAIR	WM disease	/	0.81	0.75		0.83	
(Yang et al., 2010)	U		Mild/moderate dementia	/	0.81				
(Wang et al., 2012)	U	T1, T2 and FLAIR	Aging cohort (lesions and infarcts)	/	0.77	0.70		0.80	0.83
(de Boer et al., 2009)	U	T1, PD, and FLAIR	Rotterdam study - healthy controls	/	0.72				
(Samaille et al., 2012)	U	T1 and FLAIR	MCI, CADASIL	0.96	0.72				
(Seghier et al., 2008)	U	T1	HC, stroke	/	0.64				
(Ong et al., 2012)	U	T1 and FLAIR	HC	/	0.47	0.36	0.56	0.71	
(Kawata et al., 2010)	SA	T1 and FLAIR	SVD	/	0.77				

*Direct comparison*									
BIANCA [1] (T1w space, threshold = 0.9)	S	multimodal (tested onT1 and FLAIR)	Neurodegenerative dataset 1)	0.990	0.75	0.69	0.66	0.68	0.80
BIANCA [1] (FLAIR space, threshold = 0.95)	S	multimodal (tested onT1 and FLAIR)	Neurodegenerative dataset 1)	0.989	0.79	0.75	0.71	0.73	0.82
LGA (kappa 0.2)	U	T1 and FLAIR	Neurodegenerative dataset 1)	0.852	0.69	0.67	0.69	0.60	0.72
LPA	U	FLAIR	Neurodegenerative dataset 1)	0.933	0.76	0.71	0.73	0.53	0.82
CASCADE (threshold 0.8)	U	T1, PD, and FLAIR (tested on T1 and FLAIR)	Neurodegenerative dataset 1)	0.447	0.26	0.07	0.16	0.20	0.33

a Legend: S = Supervised; U = Unsupervised; SA = Semi-Automated;

b Approximate definitions of WMH load intervals. For specific interval definitions, please refer to the single studies. For the direct comparison WMH load intervals are relative to the volume of the manual mask in the reference space (T1w).

## Footnotes

1 Different definitions of FPR exist in literature. This definition is also referred as false discovery rate (FDR), equivalent to 1-PPV (PPV = positive predictive value). (Benjamini and Hochberg, 1995. Controlling the False Discovery Rate - a Practical and Powerful Approach to Multiple Testing. Journal of the Royal Statistical Society Series B-Methodological 57, 289–300.)
