# Supplementary material for: BIANCA (Brain Intensity AbNormality Classification Algorithm): A new tool for automated segmentation of white matter hyperintensities
Source: Neuroimage. 2016 Nov 1;141:191–205. doi: 10.1016/j.neuroimage.2016.07.018 (PMC5035138; doi:10.1016/j.neuroimage.2016.07.018)
Supplement: Supplementary file 1 — Supplementary material [file mmc1.docx]

**Supplementary material**

**BIANCA (Brain Intensity AbNormality Classification Algorithm): a new tool for automated segmentation of white matter hyperintensities**

1. **Methods**
   1. **BIANCA optimization – phase II**

In the second phase of BIANCA optimisation we started from the combination of the best values for each option found in the first phase (BIANCA[1]: FLAIR+T1 registered in T1 space, sw=1, no patch, high WMH load training subjects, no border location for non-WMH training points, FU number of training points, threshold=0.9, exclusion mask applied), and repeated the process of changing one option at a time (see section *2.3 BIANCA optimisation*), to test if this was in fact the optimal solution.

If the parameter/option settings turn out not to be the optimal solution (i.e. not a stable maximum) then this phase is repeated, using the set of options corresponding to the newly found maximum as a new starting point, until a set is found that is optimal. This essentially implements a local, uphill optimisation method. More sophisticated optimisation strategies could also be employed, but in our case this simple method was sufficient to find a stable maximum that resulted in good overall segmentation performance in both datasets without iteration. For other datasets it may be necessary to iterate this, which in most cases is a relatively low computational overhead as only a subset of subjects are used for this evaluation.

In particular, we re-evaluated the different options as follows:

1. Multiple MRI modalities and exclusion mask. Values tested: FLAIR only, FLAIR+T1, FLAIR only + exclusion mask applied, FLAIR+T1 + exclusion mask applied. In addition we tested the effect of using bias field corrected T1 instead of the original image (FLAIR+T1 bias corrected + exclusion mask applied). Although it is a highly recommended step for most applications, is not clear to us that bias correction methods would always be beneficial in this context. In fact, bias field correction approaches often rely on tissue-type segmentations or assumptions about the image intensities that are inaccurate in the presence of WMH. Hence it is equally possible that application of bias correction methods may decrease or increase the accuracy of the WMH segmentation.
2. Threshold optimisation. Values tested: 0.8, 0.85, 0.9, 0.95, 0.99.
3. Spatial weighting. Values tested: sw=1, sw=5, sw=10.
4. Patch. As shown in the first phase, the use of a patch with a small kernel (D=3) produced an equivalent optimal solution to the chosen one. Therefore, this option was not further tested in the second phase.
5. Subjects included in the training set. Values tested: 21 subjects with no restriction on WMH load, 11 (any WMH load) subjects with high WMH load or 10 subjects with low WMH load.
6. Location of non-WMH training points. As shown in the first phase, the use of either any location for non-WMH or avoiding the lesion’s edge produced similar results. However, when combining the best options (Figure 2, panel H) the exclusion of the lesion’s edge (no border) gave the best results. Therefore, this option was not further tested in the second phase.
7. Number (and location) of training points. Given the increased performance using an unbalanced number of training points for the WMH and non-WMH classes option, we did not further test the FE and AE options. Instead, we tested the effect of increasing the number of points either maintaining the same ratio (1:5) between the two classes (up to 10000 WMH class and 50000 non-WMH class) or increasing only the number of training points for the non-WMH class (up to 2000 WMH class and 58000 non-WMH class – 1:29 ratio, to maintain the same total number of 60000 training points for the two additional tests)
   1. **Choice of reference modality**

All the MRI modalities used by BIANCA need to be registered to a consistent reference MRI modality, but BIANCA is flexible in terms of which modality to use as reference. In this study we decided to use T1 images as reference for the main analyses on Dataset 1, to avoid down-sampling any other image. However, it is possible that the registration and interpolation of the FLAIR image, where WMH are more readily detectable, could influence BIANCA performance. For this reason we repeated the analyses with a sub-set of options also on data registered in FLAIR space and compared the results to the corresponding option in T1 space (the measures of overlap were calculated with respect to the manual mask in the corresponding reference space). In particular we tested:

- Default option (FLAIR+T1, sw=1, no patch, any WMH load training subjects, any location for non-WMH training points, FE number of training points, exclusion mask applied) in FLAIR space (with threshold optimisation) with respect to T1 space (threshold = 0.95)
- Different location of non-WMH training points (any, no border, surround) in FLAIR space with respect to T1 space (any, threshold = 0.95), to test if the results obtained in T1 space were due to the fact that FLAIR images were registered to T1, possibly introducing interpolation errors at the lesion’s edge.
- Best option found in T1 space (BIANCA[1]: FLAIR+T1, sw=1, no patch, high WMH load training subjects, no border location for non-WMH training points, FU number of training points, exclusion mask applied) in FLAIR space (with threshold optimisation) with respect to T1 space (threshold=0.9)
  1. **Direct comparison with existing approaches**

We performed a direct comparison on Dataset 1 between BIANCA and three freely available algorithms: CASCADE (Damangir et al., 2012)(ki.se/en/nvs/cascade), and the toolbox “LST: Lesion Segmentation Tool” (http://www.applied-statistics.de/lst.html) (Schmidt et al., 2012), using its two available algorithms: LGA (lesion growth algorithm) and LPA (lesion prediction algorithm).

CASCADE is an unsupervised segmentation method based on a cascade of support vector machine with active learning. It is flexible in terms of multiple MRI modalities as input (any combination of T1, T2, FLAIR and proton density) and uses FLAIR as reference image. We ran it on T1w and FLAIR images, after performing the recommended preprocessing pipeline for T1-wieghted images, which includes T1 brain extraction, bias field correction and tissue segmentation using BET and FAST. Similarly to BIANCA, the output of CASCADE needs thresholding (in this case it is a p-values map), therefore we calculated measures of overlap and the volumetric agreement with the manual mask (in T1w space) for several thresholds to select the optimal one.

LST is an open source toolbox for SPM with two unsupervised algorithms implemented for lesion segmentation. The first, a lesion growth algorithm (LGA) (Schmidt et al., 2012), requires a T1w and a FLAIR image and uses T1w as reference space. It combines the information obtained from the segmentation of T1 image into the three tissue classes with the FLAIR intensities in order to calculate lesion belief maps. These maps are thresholded by a pre-chosen initial threshold (kappa) forming an initial binary lesion map, which is subsequently grown along voxels that appear hyperintense in the FLAIR image. The result is a lesion probability map in T1w space. As suggested by the developers, we tested different kappa values from 0.05 to 0.9 and compared the obtained probability map thresholded at 0.5 with the manual mask (in T1w space), to select the optimal kappa for our dataset and compare LGA performance against BIANCA.

The second algorithm, a lesion prediction algorithm (LPA), requires a FLAIR image only. LPA was trained by a logistic regression model with the data of 53 MS patients. Binary lesion maps of these patients were used as response values. As covariates a similar lesion belief map as for LGA is used as well as a spatial covariate that takes into account voxel specific changes in lesion probability. Regression HD (http://www.applied-statistics.de/RegressionHD_en.html) is then used for fitting large-scale regression models. The parameters of this model fit are then used to segment lesions in new images by providing an estimate for the lesion probability for each voxel. Similarly for LGA, we evaluated LPA performance comparing the obtained probability map thresholded at 0.5 with the manual masks (in FLAIR space).

After finding the optimal threshold for CASCADE, and the optimal kappa value for LGA, we compared the performance of the three algorithms (in terms of overlap and volumetric agreement with the manual masks) against the optimal results from BIANCA.

1. **Results and discussion**
   1. **BIANCA optimization – phase II**

Results of the second optimisation phase are shown in supplementary figure S3 and supplementary Table S2. In particular, we observed that:

1. Multiple MRI modalities and exclusion mask. All the tested options gave similar results in terms of SI, ICC and FNR cluster-level. Also the use of T1 images after applying bias field correction gave very similar results, suggesting that this processing step does not have a big impact on the results. (Figure S3.A)
2. Threshold optimisation. The threshold of 0.9 still gave the best results in terms of SI and ICC. An alternative optimal solution was obtained using a threshold of 0.85 (same SI and ICC, higher FPR and lower FNR) (Figure S3.B).
3. Spatial weighting. The spatial weighting giving the best results was still sw=1 (Figure S3.C).
4. Subjects included in the training set. The use of subjects with high WMH load still gave the best results in terms of SI and ICC. An alternative optimal solution was obtained using a all 21 subjects for the training (any WMH load option) (same SI, ICC and FNR) (Figure S3.D).
5. Number (and location) of training points. Both when increasing the number of training points for both classes (Figure S3.E) and increasing only the number of non-WMH training points (Figure S3.F), the results showed similar performance to the optimal settings found with lower number of training points (same SI, similar ICC and FNR). Alternative optimal solutions were obtained using 10000 points for WMH class and 50000 for non-WMH class, threshold 0.9, or 2000 points for WMH class and 58000 points for non-WMH class, with threshold 0.5.

The second optimisation phase confirmed that the chosen values for each option were still the optimal ones (giving comparable or higher performance to the tested alternatives) when varying them. Although further tests using different combination of number of points and ratio between the two classes are needed, this suggests that BIANCA segmentation is already accurate when using a relatively small number of training points.

- 1. **Choice of reference modality**

Supplementary Figure S3 (panels G,H,I) and supplementary Table S2 show the results of the analyses on data registered in FLAIR space and compared the results to the corresponding best option in T1 space.

- Using the default set of options in FLAIR space (Figure S3.G) resulted in higher SI, higher ICC but higher FNR with respect to the same setting on images registered in T1 space, giving an alternative optimal solution.
- When comparing the different locations for non-WMH training points, the surround option still gave the worst performance (Figure S3.H), suggesting that avoiding voxels at the lesion’s edge is generally beneficial, even when no registration or interpolation is performed on the FLAIR image.
- When comparing the use of the two reference modalities using the optimal set of options found in T1 space, the use of images registered in FLAIR space (Figure S3.I) still gave better SI, but lower ICC and higher FNR.

Although further evaluations are needed, the additional tests presented here suggest that the choice of the reference modality does not have a big impact on the results.

**Supplementary tables**

**Table S1.** BIANCA optimisation phase I (Dataset 1).

| **option** | **SI** | **FPR** | **FNR** | **FPR clusters** | **FNR clusters** | **DER** | **OER** | **ICC** |
| --- | --- | --- | --- | --- | --- | --- | --- | --- |
| **A) Multimodal and masking** |  |  |  |  |  |  |  |  |
| FLAIR | 0.65 | 0.45 | 0.14 | 0.97 | 0.01 | 0.17 | 0.53 | 0.931 |
| FLAIR + T1 | 0.67 | 0.41 | 0.15 | 0.95 | 0.01 | 0.11 | 0.54 | 0.936 |
| FLAIR + MASK | 0.70 | 0.38 | 0.16 | 0.91 | 0.01 | 0.05 | 0.55 | 0.971 |
| ***FLAIR + T1 + MASK*** | 0.70 | 0.37 | 0.18 | 0.89 | 0.01 | 0.05 | 0.56 | 0.966 |
| **B) Threshold selection** |  |  |  |  |  |  |  |  |
| threshold=0.8 | 0.57 | 0.55 | 0.10 | 0.96 | 0.01 | 0.06 | 0.79 | 0.778 |
| threshold=0.85 | 0.61 | 0.51 | 0.12 | 0.94 | 0.01 | 0.05 | 0.73 | 0.847 |
| threshold=0.9 | 0.65 | 0.45 | 0.14 | 0.93 | 0.01 | 0.05 | 0.64 | 0.911 |
| ***threshold=0.95*** | 0.70 | 0.37 | 0.18 | 0.89 | 0.01 | 0.05 | 0.56 | 0.966 |
| threshold=0.99 | 0.70 | 0.23 | 0.34 | 0.79 | 0.04 | 0.04 | 0.56 | 0.977 |
| **C) Spatial weighting** |  |  |  |  |  |  |  |  |
| ***sw=1*** | 0.70 | 0.37 | 0.18 | 0.89 | 0.01 | 0.05 | 0.56 | 0.996 |
| sw=5 | 0.63 | 0.43 | 0.24 | 0.83 | 0.10 | 0.03 | 0.71 | 0.961 |
| sw=10 | 0.51 | 0.56 | 0.30 | 0.85 | 0.26 | 0.03 | 0.96 | 0.839 |
| **D) Patch** |  |  |  |  |  |  |  |  |
| ***none*** | 0.70 | 0.37 | 0.18 | 0.89 | 0.01 | 0.05 | 0.56 | 0.996 |
| ***D=3*** | 0.71 | 0.36 | 0.17 | 0.84 | 0.01 | 0.05 | 0.52 | 0.97 |
| D=6 | 0.71 | 0.36 | 0.19 | 0.83 | 0.02 | 0.04 | 0.55 | 0.968 |
| D=9 | 0.71 | 0.35 | 0.20 | 0.84 | 0.02 | 0.04 | 0.55 | 0.972 |
| **E) WMH load of training subjects** | |  |  |  |  |  |  |  |
| ***anyWMH*** | 0.70 | 0.37 | 0.18 | 0.89 | 0.01 | 0.05 | 0.56 | 0.996 |
| lowWMH | 0.67 | 0.42 | 0.17 | 0.90 | 0.02 | 0.05 | 0.61 | 0.944 |
| ***highWMH*** | 0.72 | 0.31 | 0.22 | 0.87 | 0.02 | 0.05 | 051 | 0.986 |
| **F) Location of non-WMH training points** | |  |  |  |  |  |  |  |
| ***any*** | 0.70 | 0.37 | 0.18 | 0.89 | 0.01 | 0.05 | 0.56 | 0.996 |
| ***no border*** | 0.69 | 0.40 | 0.17 | 0.90 | 0.01 | 0.05 | 0.58 | 0.954 |
| surround | 0.57 | 0.06 | 0.58 | 0.42 | 0.15 | 0.03 | 0.83 | 0.814 |
| **G) Number of training points (plus threshold optimisation)** | | | |  |  |  |  |  |
| G1) FE any (same as B, optimal threshold 0.95) | | |  |  |  |  |  |  |
| ***threshold=0.95*** | 0.70 | 0.37 | 0.18 | 0.89 | 0.01 | 0.05 | 0.56 | 0.966 |
| G2) FE noborder |  |  |  |  |  |  |  |  |
| threshold=0.8 | 0.57 | 0.56 | 0.10 | 0.96 | 0.01 | 0.06 | 0.81 | 0.760 |
| threshold=0.85 | 0.60 | 0.52 | 0.11 | 0.95 | 0.01 | 0.06 | 0.74 | 0.830 |
| threshold=0.9 | 0.64 | 0.47 | 0.13 | 0.93 | 0.01 | 0.06 | 0.66 | 0.895 |
| threshold=0.95 | 0.69 | 0.40 | 0.17 | 0.90 | 0.01 | 0.05 | 0.58 | 0.954 |
| ***threshold=0.99*** | 0.73 | 0.26 | 0.27 | 0.82 | 0.04 | 0.04 | 0.51 | 0.989 |
| G3) AE any |  |  |  |  |  |  |  |  |
| threshold=0.8 | 0.59 | 0.53 | 0.10 | 0.96 | 0.01 | 0.07 | 0.75 | 0.815 |
| threshold=0.85 | 0.62 | 0.49 | 0.12 | 0.95 | 0.01 | 0.06 | 0.69 | 0.873 |
| threshold=0.9 | 0.66 | 0.44 | 0.14 | 0.94 | 0.01 | 0.06 | 0.61 | 0.927 |
| threshold=0.95 | 0.70 | 0.36 | 0.19 | 0.91 | 0.01 | 0.05 | 0.54 | 0.973 |
| ***threshold=0.99*** | 0.71 | 0.22 | 0.34 | 0.82 | 0.02 | 0.04 | 0.55 | 0.973 |
| G4) AE noborder |  |  |  |  |  |  |  |  |
| threshold=0.8 | 0.57 | 0.56 | 0.09 | 0.97 | 0.01 | 0.06 | 0.79 | 0.778 |
| threshold=0.85 | 0.60 | 0.52 | 0.11 | 0.96 | 0.01 | 0.06 | 0.73 | 0.837 |
| threshold=0.9 | 0.64 | 0.47 | 0.12 | 0.94 | 0.01 | 0.06 | 0.66 | 0.894 |
| threshold=0.95 | 0.69 | 0.40 | 0.16 | 0.92 | 0.01 | 0.05 | 0.58 | 0.951 |
| ***threshold=0.99*** | 0.72 | 0.27 | 0.27 | 0.86 | 0.02 | 0.04 | 0.52 | 0.989 |
| G5) FU any |  |  |  |  |  |  |  |  |
| threshold=0.8 | 0.73 | 0.31 | 0.19 | 0.83 | 0.01 | 0.03 | 0.50 | 0.983 |
| threshold=0.85 | 0.75 | 0.27 | 0.22 | 0.80 | 0.01 | 0.03 | 0.48 | 0.989 |
| ***threshold=0.9*** | 0.75 | 0.23 | 0.26 | 0.75 | 0.03 | 0.03 | 0.47 | 0.989 |
| threshold=0.95 | 0.73 | 0.17 | 0.33 | 0.67 | 0.06 | 0.02 | 0.51 | 0.971 |
| threshold=0.99 | 0.62 | 0.09 | 0.52 | 0.47 | 0.14 | 0.03 | 0.72 | 0.859 |
| G6) FU noborder |  |  |  |  |  |  |  |  |
| threshold=0.8 | 0.72 | 0.34 | 0.17 | 0.85 | 0.01 | 0.03 | 0.52 | 0.974 |
| threshold=0.85 | 0.74 | 0.31 | 0.19 | 0.82 | 0.02 | 0.03 | 0.50 | 0.984 |
| ***threshold=0.9*** | 0.75 | 0.27 | 0.22 | 0.79 | 0.02 | 0.03 | 0.48 | 0.990 |
| threshold=0.95 | 0.75 | 0.21 | 0.27 | 0.73 | 0.04 | 0.02 | 0.48 | 0.987 |
| threshold=0.99 | 0.69 | 0.13 | 0.41 | 0.60 | 0.11 | 0.02 | 0.59 | 0.936 |
| Comparison of options after threshold optimisation | | |  |  |  |  |  |  |
| FE any, thr=0.95 | 0.70 | 0.37 | 0.18 | 0.89 | 0.01 | 0.05 | 0.56 | 0.966 |
| FE noborder, thr=0.99 | 0.73 | 0.26 | 0.27 | 0.82 | 0.04 | 0.04 | 0.51 | 0.989 |
| AE any, thr=0.99 | 0.71 | 0.22 | 0.34 | 0.82 | 0.02 | 0.04 | 0.55 | 0.973 |
| AE no border, thr=0.99 | 0.72 | 0.27 | 0.27 | 0.86 | 0.02 | 0.04 | 0.52 | 0.989 |
| ***FU any, thr=0.9*** | 0.75 | 0.23 | 0.26 | 0.75 | 0.03 | 0.03 | 0.47 | 0.989 |
| ***FU no border, thr=0.9*** | 0.75 | 0.27 | 0.22 | 0.79 | 0.02 | 0.03 | 0.48 | 0.990 |
| **H) Combination of best options** |  |  |  |  |  |  |  |  |
| (a) highWMH, FU, any, mask | 0.74 | 0.17 | 0.32 | 0.70 | 0.03 | 0.03 | 0.50 | 0.979 |
| ***(b) highWMH, FU, no border, mask [1]*** | 0.75 | 0.22 | 0.26 | 0.77 | 0.02 | 0.03 | 0.47 | 0.990 |
| (c) highWMH, FU, any, patch D=3, mask | 0.75 | 0.16 | 0.31 | 0.60 | 0.05 | 0.02 | 0.48 | 0.979 |
| ***(d) highWMH, FU, no border, patch D=3, mask [2]*** | 0.76 | 0.22 | 0.25 | 0.67 | 0.03 | 0.03 | 0.46 | 0.990 |

***Bold and italicised*** options were chosen as best options for the specific optimisation step. [1] and [2] were selected as final best options. Legend: SI=Dice similarity index; FPR=false positive ratio; FNR=false negative ratio; DER=detection error rate; OER=outline error rate; ICC=intra class correlation coefficient; FE=fixed equal; AE= all equal; FU= fixed unbalanced. Please refer to the main text for further details.

**Table S2.** BIANCA optimisation phase II (Dataset 1).

| **option** | **SI** | **FPR** | **FNR** | **FPR clusters** | **FNR clusters** | **DER** | **OER** | **ICC** |
| --- | --- | --- | --- | --- | --- | --- | --- | --- |
| **A) Multimodal and masking on BIANCA [1]** | |  |  |  |  |  |  |  |
| FLAIR | 0.75 | 0.28 | 0.20 | 0.91 | 0.02 | 0.07 | 0.43 | 0.988 |
| FLAIR + T1 | 0.75 | 0.23 | 0.24 | 0.82 | 0.02 | 0.04 | 0.45 | 0.989 |
| FLAIR + mask | 0.75 | 0.26 | 0.22 | 0.80 | 0.02 | 0.03 | 0.46 | 0.992 |
| *BIANCA [1] - FLAIR + T1 + mask* | 0.75 | 0.22 | 0.26 | 0.77 | 0.02 | 0.03 | 0.47 | 0.990 |
| ***FLAIR + T1(BIASCORR) + mask*** | 0.75 | 0.22 | 0.25 | 0.78 | 0.02 | 0.03 | 0.46 | 0.991 |
| **B) Threshold selection on BIANCA [1]** | |  |  |  |  |  |  |  |
| threshold=0.8 | 0.74 | 0.30 | 0.20 | 0.83 | 0.01 | 0.03 | 0.49 | 0.985 |
| ***threshold=0.85*** | 0.75 | 0.26 | 0.23 | 0.81 | 0.01 | 0.03 | 0.47 | 0.990 |
| *BIANCA [1] - threshold=0.9* | 0.75 | 0.22 | 0.26 | 0.77 | 0.02 | 0.03 | 0.47 | 0.990 |
| threshold=0.95 | 0.74 | 0.17 | 0.32 | 0.71 | 0.03 | 0.02 | 0.49 | 0.980 |
| threshold=0.99 | 0.66 | 0.10 | 0.47 | 0.52 | 0.11 | 0.02 | 0.66 | 0.905 |
| **C) Spatial weighting on BIANCA [1]** |  |  |  |  |  |  |  |  |
| *BIANCA [1] - sw=1* | 0.75 | 0.22 | 0.26 | 0.77 | 0.02 | 0.03 | 0.47 | 0.990 |
| sw=5 | 0.72 | 0.23 | 0.31 | 0.59 | 0.18 | 0.03 | 0.53 | 0.959 |
| sw=10 | 0.62 | 0.33 | 0.38 | 0.49 | 0.33 | 0.03 | 0.72 | 0.865 |
| **D) WMH load of training subjects on BIANCA [1]** | | |  |  |  |  |  |  |
| ***any WMH*** | 0.75 | 0.27 | 0.22 | 0.79 | 0.02 | 0.03 | 0.48 | 0.990 |
| low WMH | 0.73 | 0.29 | 0.22 | 0.80 | 0.05 | 0.03 | 0.50 | 0.989 |
| *BIANCA [1] - high WMH* | 0.75 | 0.22 | 0.26 | 0.77 | 0.02 | 0.03 | 0.47 | 0.990 |
| **E) Number of training points - FU - Increased both WMH and non-WMH (10000 WMH - 50000 non-WMH)** | | | | | | | | |
| *BIANCA [1] - FU (2000 WMH, 10000 non-WMH)* | 0.75 | 0.22 | 0.26 | 0.77 | 0.02 | 0.03 | 0.47 | 0.990 |
| threshold=0.4 | 0.60 | 0.53 | 0.09 | 0.95 | 0.01 | 0.05 | 0.75 | 0.825 |
| threshold=0.5 | 0.64 | 0.48 | 0.11 | 0.94 | 0.01 | 0.04 | 0.68 | 0.883 |
| threshold=0.6 | 0.67 | 0.43 | 0.13 | 0.91 | 0.01 | 0.05 | 0.60 | 0.926 |
| threshold=0.7 | 0.70 | 0.38 | 0.15 | 0.89 | 0.01 | 0.04 | 0.55 | 0.958 |
| threshold=0.8 | 0.73 | 0.32 | 0.18 | 0.84 | 0.01 | 0.03 | 0.51 | 0.980 |
| ***threshold=0.9*** | 0.75 | 0.25 | 0.24 | 0.78 | 0.02 | 0.03 | 0.47 | 0.991 |
| threshold=0.95 | 0.74 | 0.20 | 0.30 | 0.73 | 0.05 | 0.03 | 0.49 | 0.984 |
| threshold=0.99 | 0.67 | 0.12 | 0.44 | 0.55 | 0.07 | 0.02 | 0.63 | 0.921 |
| **F) Number of training points - FU - Increased non-WMH 2000 WMH - 58000 non-WMH)** | | | | | | |  |  |
| *BIANCA [1] - FU (2000 WMH, 10000 non-WMH)* | 0.75 | 0.22 | 0.26 | 0.77 | 0.02 | 0.03 | 0.47 | 0.990 |
| threshold=0.4 | 0.74 | 0.30 | 0.20 | 0.82 | 0.01 | 0.03 | 0.49 | 0.985 |
| ***threshold=0.5*** | 0.75 | 0.25 | 0.23 | 0.79 | 0.01 | 0.03 | 0.47 | 0.991 |
| threshold=0.6 | 0.75 | 0.21 | 0.26 | 0.74 | 0.02 | 0.03 | 0.47 | 0.990 |
| threshold=0.7 | 0.75 | 0.17 | 0.30 | 0.69 | 0.05 | 0.03 | 0.48 | 0.982 |
| threshold=0.8 | 0.73 | 0.14 | 0.36 | 0.61 | 0.08 | 0.02 | 0.52 | 0.965 |
| threshold=0.9 | 0.68 | 0.09 | 0.44 | 0.50 | 0.11 | 0.02 | 0.61 | 0.925 |
| threshold=0.95 | 0.64 | 0.07 | 0.50 | 0.41 | 0.15 | 0.02 | 0.70 | 0.877 |
| threshold=0.99 | 0.51 | 0.04 | 0.64 | 0.25 | 0.22 | 0.05 | 0.93 | 0.737 |
| **G) FLAIR space - default options** |  |  |  |  |  |  |  |  |
| *T1 space - default options - thr=0.95* | 0.70 | 0.37 | 0.18 | 0.89 | 0.01 | 0.05 | 0.56 | 0.966 |
| FLAIR space - threshold=0.8 | 0.60 | 0.53 | 0.05 | 0.93 | 0.02 | 0.11 | 0.68 | 0.843 |
| FLAIR space - threshold=0.85 | 0.64 | 0.49 | 0.06 | 0.91 | 0.02 | 0.10 | 0.62 | 0.882 |
| FLAIR space - threshold=0.9 | 0.67 | 0.45 | 0.07 | 0.88 | 0.02 | 0.09 | 0.56 | 0.922 |
| ***FLAIR space - threshold=0.95*** | 0.72 | 0.38 | 0.10 | 0.84 | 0.02 | 0.08 | 0.49 | 0.961 |
| FLAIR space - threshold=0.99 | 0.74 | 0.25 | 0.24 | 0.71 | 0.07 | 0.05 | 0.46 | 0.984 |
| **H) FLAIR space - location of non-WMH points** | | |  |  |  |  |  |  |
| *T1 space - default options - thr=0.95 - any* | 0.70 | 0.37 | 0.18 | 0.89 | 0.01 | 0.05 | 0.56 | 0.966 |
| ***FLAIR space - default options - thr=0.95 - any*** | 0.72 | 0.38 | 0.10 | 0.84 | 0.02 | 0.08 | 0.49 | 0.961 |
| FLAIR space - default options - thr=0.95 - no border | 0.70 | 0.41 | 0.09 | 0.87 | 0.02 | 0.08 | 0.51 | 0.946 |
| FLAIR space - default options - thr=0.95 - surround | 0.66 | 0.10 | 0.47 | 0.46 | 0.16 | 0.05 | 0.64 | 0.940 |
| **I) T2space - best option BIANCA[1]** |  |  |  |  |  |  |  |  |
| *BIANCA [1]* | 0.75 | 0.22 | 0.26 | 0.77 | 0.02 | 0.03 | 0.47 | 0.990 |
| FLAIR space - BIANCA [1] - threshold=0.8 | 0.74 | 0.35 | 0.10 | 0.81 | 0.02 | 0.06 | 0.45 | 0.970 |
| FLAIR space - BIANCA [1] - threshold=0.85 | 0.76 | 0.31 | 0.12 | 0.78 | 0.03 | 0.06 | 0.42 | 0.979 |
| FLAIR space - BIANCA [1] - threshold=0.9 | 0.78 | 0.27 | 0.14 | 0.75 | 0.04 | 0.05 | 0.39 | 0.986 |
| ***FLAIR space - BIANCA [1] - threshold=0.95*** | 0.79 | 0.22 | 0.19 | 0.68 | 0.06 | 0.04 | 0.38 | 0.989 |
| FLAIR space - BIANCA [1] - threshold=0.99 | 0.74 | 0.14 | 0.33 | 0.51 | 0.14 | 0.04 | 0.48 | 0.951 |

*Italicised* option is the optimal solution found in the first optimisation phase. ***Bold and italicised*** options were optimal alternative options for the specific optimisation step. Legend: SI=Dice similarity index; FPR=false positive ratio; FNR=false negative ratio; DER=detection error rate; OER=outline error rate; ICC=intra class correlation coefficient; FE=fixed equal; AE= all equal; FU= fixed unbalanced. Please refer to the main text for further details.

**Table S3.** BIANCA optimisation (Dataset 2).

| **option** | **SI** | **FPR** | **FNR** | **FPR clusters** | **FNR clusters** | **DER** | **OER** | **ICC** |
| --- | --- | --- | --- | --- | --- | --- | --- | --- |
| **A) Threshold selection and masking** |  |  |  |  |  |  |  |  |
| threshold=0.8 | 0.31 | 0.78 | 0.25 | 0.95 | 0.14 | 0.31 | 1.06 | 0.526 |
| threshold=0.85 | 0.35 | 0.74 | 0.28 | 0.94 | 0.16 | 0.33 | 0.97 | 0.662 |
| threshold=0.9 | 0.39 | 0.69 | 0.32 | 0.92 | 0.19 | 0.34 | 0.88 | 0.800 |
| threshold=0.95 | 0.44 | 0.62 | 0.39 | 0.89 | 0.23 | 0.33 | 0.79 | 0.909 |
| ***threshold=0.99*** | 0.46 | 0.45 | 0.56 | 0.78 | 0.35 | 0.30 | 0.78 | 0.899 |
| threshold=0.99 - unmasked | 0.43 | 0.55 | 0.53 | 0.87 | 0.32 | 0.46 | 0.67 | 0.926 |
| **B) 2D patch** |  |  |  |  |  |  |  |  |
| none | 0.46 | 0.45 | 0.56 | 0.78 | 0.35 | 0.30 | 0.78 | 0.899 |
| 2D patch D=3 | 0.47 | 0.50 | 0.52 | 0.81 | 0.31 | 0.35 | 0.71 | 0.878 |
| **C) Location non-WMH points** |  |  |  |  |  |  |  |  |
| *any* | 0.46 | 0.45 | 0.56 | 0.78 | 0.35 | 0.30 | 0.78 | 0.899 |
| ***no border*** | 0.48 | 0.48 | 0.52 | 0.79 | 0.33 | 0.27 | 0.78 | 0.912 |
| surround | 0.14 | 0.18 | 0.92 | 0.31 | 0.74 | 0.47 | 1.25 | 0.334 |
| **Number of training points (plus threshold optimisation)** | | | |  |  |  |  |  |
| D) FE – no border - threshold selection | |  |  |  |  |  |  |  |
| threshold=0.8 | 0.31 | 0.78 | 0.24 | 0.95 | 0.13 | 0.30 | 1.08 | 0.507 |
| threshold=0.85 | 0.35 | 0.75 | 0.27 | 0.94 | 0.15 | 0.32 | 0.98 | 0.646 |
| threshold=0.9 | 0.39 | 0.70 | 0.31 | 0.92 | 0.18 | 0.33 | 0.90 | 0.787 |
| threshold=0.95 | 0.43 | 0.63 | 0.38 | 0.89 | 0.23 | 0.30 | 0.83 | 0.898 |
| ***threshold=0.99*** | 0.48 | 0.48 | 0.52 | 0.79 | 0.33 | 0.27 | 0.78 | 0.912 |
| E) AE – no border - threshold selection | |  |  |  |  |  |  |  |
| threshold=0.8 | 0.29 | 0.80 | 0.24 | 0.96 | 0.12 | 0.28 | 1.15 | 0.416 |
| threshold=0.85 | 0.32 | 0.77 | 0.27 | 0.95 | 0.13 | 0.33 | 1.02 | 0.578 |
| threshold=0.9 | 0.37 | 0.72 | 0.31 | 0.94 | 0.16 | 0.35 | 0.91 | 0.754 |
| threshold=0.95 | 0.43 | 0.64 | 0.38 | 0.91 | 0.20 | 0.36 | 0.79 | 0.894 |
| ***threshold=0.99*** | 0.46 | 0.48 | 0.54 | 0.82 | 0.30 | 0.32 | 0.75 | 0.914 |
| F) FU – no border - threshold selection | |  |  |  |  |  |  |  |
| threshold=0.8 | 0.49 | 0.55 | 0.37 | 0.83 | 0.24 | 0.20 | 0.81 | 0.929 |
| threshold=0.85 | 0.51 | 0.51 | 0.40 | 0.80 | 0.26 | 0.19 | 0.79 | 0.936 |
| **threshold=0.9** | 0.52 | 0.46 | 0.45 | 0.76 | 0.30 | 0.19 | 0.76 | 0.933 |
| threshold=0.95 | 0.52 | 0.39 | 0.51 | 0.69 | 0.36 | 0.20 | 0.76 | 0.909 |
| *threshold=0.99* | 0.46 | 0.26 | 0.65 | 0.54 | 0.48 | 0.21 | 0.87 | 0.808 |
| **G) Comparison of options after threshold optimisation** | | |  |  |  |  |  |  |
| FE no border, thr=0.99 | 0.48 | 0.48 | 0.52 | 0.79 | 0.33 | 0.27 | 0.78 | 0.912 |
| AE no border, thr=0.99 | 0.46 | 0.48 | 0.54 | 0.82 | 0.30 | 0.32 | 0.75 | 0.914 |
| ***FU no border, thr=0.9*** | 0.52 | 0.46 | 0.45 | 0.76 | 0.30 | 0.19 | 0.76 | 0.933 |
| **H) Combination of best options** |  |  |  |  |  |  |  |  |
| ***(a) FU no border, thr=0.9, no patch, mask [§]*** | 0.52 | 0.46 | 0.45 | 0.76 | 0.30 | 0.19 | 0.76 | 0.919 |
| (b) FU no border, thr=0.9, 2D patch D=3, mask | 0.51 | 0.50 | 0.41 | 0.79 | 0.26 | 0.26 | 0.72 | 0.933 |

***Bold and italicised*** options were chosen as best options for the specific optimisation step. [§] was selected as final best options. Legend: SI=Dice similarity index; FPR=false positive ratio; FNR=false negative ratio; DER=detection error rate; OER=outline error rate; ICC=intra class correlation coefficient; FE=fixed equal; AE= all equal; FU= fixed unbalanced. Please refer to the main text for further details.

**Table S4.** Direct comparison with available tools.

| **option** | **SI** | **FPR** | **FNR** | **FPR clusters** | **FNR clusters** | **DER** | **OER** | **ICC** |
| --- | --- | --- | --- | --- | --- | --- | --- | --- |
| **BIANCA [1]** |  |  |  |  |  |  |  |  |
| T1 space thr=0.9 | 0.75 | 0.22 | 0.26 | 0.77 | 0.02 | 0.03 | 0.47 | 0.990 |
| FLAIR space thr=0.95 | 0.79 | 0.22 | 0.19 | 0.68 | 0.06 | 0.04 | 0.38 | 0.989 |
| **LGA** (T1w space) | | | | | | | | |
| kappa=0.05 | 0.59 | 0.47 | 0.27 | 0.93 | 0.19 | 0.39 | 0.43 | 0.878 |
| kappa=0.1 | 0.67 | 0.32 | 0.31 | 0.82 | 0.21 | 0.19 | 0.48 | 0.887 |
| ***kappa=0.2*** | 0.69 | 0.22 | 0.36 | 0.61 | 0.26 | 0.10 | 0.52 | 0.852 |
| kappa=0.3 | 0.68 | 0.19 | 0.39 | 0.49 | 0.33 | 0.09 | 0.54 | 0.817 |
| kappa=0.4 | 0.67 | 0.17 | 0.42 | 0.42 | 0.37 | 0.08 | 0.58 | 0.773 |
| kappa=0.5 | 0.66 | 0.15 | 0.45 | 0.37 | 0.39 | 0.08 | 0.61 | 0.733 |
| kappa=0.6 | 0.64 | 0.14 | 0.48 | 0.32 | 0.42 | 0.08 | 0.64 | 0.675 |
| kappa=0.7 | 0.62 | 0.13 | 0.50 | 0.29 | 0.46 | 0.09 | 0.66 | 0.642 |
| kappa=0.8 | 0.61 | 0.12 | 0.52 | 0.26 | 0.49 | 0.10 | 0.68 | 0.621 |
| kappa=0.9 | 0.59 | 0.11 | 0.54 | 0.22 | 0.51 | 0.10 | 0.71 | 0.601 |
| **LPA**  (FLAIR space) | 0.76 | 0.29 | 0.15 | 0.63 | 0.11 | 0.09 | 0.40 | 0.933 |
| **CASCADE** (FLAIR space) | | | | | | | | |
| threshold=0.6 | 0.12 | 0.92 | 0.60 | 0.99 | 0.02 | 0.59 | 1.16 | 0.049 |
| threshold=0.7 | 0.19 | 0.85 | 0.62 | 0.98 | 0.04 | 0.75 | 0.87 | 0.242 |
| ***threshold=0.8*** | 0.26 | 0.74 | 0.66 | 0.92 | 0.07 | 0.67 | 0.81 | 0.447 |
| threshold=0.85 | 0.28 | 0.66 | 0.71 | 0.85 | 0.11 | 0.56 | 0.87 | 0.309 |
| threshold=0.9 | 0.27 | 0.56 | 0.78 | 0.73 | 0.20 | 0.42 | 1.04 | 0.118 |
| threshold=0.95 | 0.19 | 0.42 | 0.88 | 0.52 | 0.41 | 0.25 | 1.38 | 0.003 |
| threshold=0.8 + mask | 0.33 | 0.62 | 0.67 | 0.82 | 0.08 | 0.31 | 1.02 | 0.633 |

***Bold and italicised*** options is chosen as best options for comparison against BIANCA. Legend: SI=Dice similarity index; FPR=false positive ratio; FNR=false negative ratio; DER=detection error rate; OER=outline error rate; ICC=intra class correlation coefficient.

**Supplementary figure captions**

**Figure S1.** Examples of manual masks for different datasets (a,b Dataset 1; c,d Dataset 2) and WMH load (a,c low WMH load; b,d high WMH). Top row shows the FLAIR images, the bottom row shows the manual mask overlaid in blue. For Dataset 1, the corresponding T1w image is shown in the middle row.

**Figure S2.** BIANCA optimisation phase I (Dataset 1) – threshold optimisations (option G in the main text). The plots show the values of the main metrics used to evaluate BIANCA performance using different values (x axis) for the different options (please refer to the main text and supplementary table S1 for details about the options). The similarity index (SI) is shown in red, with mean value (square marker) and standard deviation (error bars) across subjects. The intra class correlation coefficient (ICC) between the total WMH volume from BIANCA output and manual segmentation is shown in blue. The black stars indicate the value(s) chosen for a specific option.

**Figure S3.** BIANCA optimisation phase II (A-F) and choice of reference modality (G-I) (Dataset 1). The plots show the values of the main metrics used to evaluate BIANCA performance using different values (x axis) for the different options (please refer to the supplementary material and supplementary Table S2 for details about the options). The similarity index (SI) is shown in red, with mean value (square marker) and standard deviation (error bars) across subjects. The intra class correlation coefficient (ICC) between the total WMH volume from BIANCA output and manual segmentation is shown in blue. The black stars indicate the optimal alternative options for the specific optimisation step.

**Figure S4.** BIANCA optimisation on Dataset 2. The plots show the values of the main metrics used to evaluate BIANCA performance using different values (x axis) for the different options (panels A-H. Please refer to the main text and supplementary Table S3 for details about the options). The similarity index (SI) is shown in red, with mean value (square marker) and standard deviation (error bars) across subjects. The intra class correlation coefficient (ICC) between the total WMH volume from BIANCA output and manual segmentation is shown in blue. The black stars indicate the value(s) chosen for a specific option. Panel I shows BIANCA performance (SI) for each subject against the WMH load (WMH volume in mL extracted from the manual masks.

Legend: FE = Fixed Equal, AE = All Equal, FU=Fixed Unbalanced number of training points; NB = no border. Panel H legend: a = high WMH load training subjects, FU training points, no patch, no border location for non-WMH training points, mask applied, threshold 0.9; b= high WMH load training subjects, FU training points, 2D patch D=3, no border location for non-WMH training points, mask applied, threshold 0.9.

**Figure S5.** Direct comparison with available tools. Panel A shows the similarity index (SI, red), with mean value (square marker) and standard deviation (error bars) across subjects and the intra class correlation coefficient (ICC, blue) between the total WMH volume from BIANCA (in T1w or in FLAIR space), CASCADE (for different thresholds), LGA (for different kappa values, threshold 0.5) and LPA (threshold 0.5) and manual segmentation. The black star indicates the optimal options for CASCADE and LGA that were chosen for comparison against BIANCA. Panel B shows volumes calculated from BIANCA, CASCADE, LGA and LPA for each subject against the WMH load (WMH volume in mL extracted from the manual masks in the reference space used by the algorithm).

**Figure S6.** Example WMH segmentation with different tools. FLAIR images from two subjects are shown, together with the manual mask and the output from the different segmentation tools either in FLAIR space or T1w space, according to the reference space used for WMH segmentation by the algorithm. CASCADE (threshold = 0.8) and LPA gave the best performance on subject number one, while subject number 2 was the one giving the best LGA (kappa = 0.2) performance. White arrows and circles highlight the main segmentation errors by CASCADE and LGA, while BIANCA and LPA show good performance in both examples.
